# Efficacy and safety of biological agents and physical therapies for delayed union or nonunion of fractures: a network meta-analysis of randomized controlled trials

**DOI:** 10.1186/s12891-026-09973-w

**Published:** 2026-05-28

**Authors:** Yage Wang, Weisheng Zhuang, Beibei Lei, Di Zhang, Shenhong Ma, Heling Wang, Qiaohua Han

**Affiliations:** 1 School of Rehabilitation Medicine, Henan University of Chinese Medicine, Zhengzhou, 450046 China; 2 Henan Provincial People’s Hospital, No.7 Weiwu Road, Jinshui District, Zhengzhou, Henan 450003 China; 3 People’s Hospital of Henan University, Zhengzhou, 450003 China

**Keywords:** Delayed union, Fracture nonunion, Biological agents, Physical therapy, Autologous cancellous bone, Bone marrow aspirate, Low-intensity pulsed ultrasound, Platelet-rich plasma

## Abstract

**Background:**

Delayed and nonunion fractures remain a significant clinical challenge in orthopedics. This study compared the effects of biologics and physical therapies for delayed or nonunion fractures.

**Methods:**

We comprehensively searched and included randomized controlled trials (RCTs) investigating biologics and physical therapies for delayed or nonunion fractures; the outcomes included healing rate, healing time and adverse events. We ranked the interventions’ therapeutic efficacy through data analysis.

**Results:**

Twenty-seven RCTs (involving 1495 patients and 15 interventions) were included. For fracture healing rate, bone marrow aspirate (BMA) combined with autologous cancellous bone (ACB) exhibited significantly better efficacy than ACB alone (OR = 8.14, 95% CI: 1.69 to 39.32). SUCRA ranking revealed that BMA + ACB (96%), platelet-rich plasma (PRP) + ACB (77.3%) and bone morphogenetic protein (BMP) + ACB (70.5%) ranked among the top three interventions for fracture healing rate. For fracture healing time, LIPUS (SMD = -9.33, 95% CI: -12.94 to -5.73) and PRP + ACB (SMD = -2.43, 95% CI: -4.14 to -0.72) both significantly reduced the fracture healing time relative to ACB. For adverse events, BMA + ACB (OR = 0.12, 95% CI: 0.03 to 0.59) and BMP (OR = 0.15, 95% CI: 0.04 to 0.61) had the lowest risk of adverse events relative to ACB alone.

**Conclusion:**

Preliminary evidence suggests that among currently available biologics and physical therapies, BMA combined with ACB may be associated with a higher fracture healing rate. LIPUS may help shorten the healing time in patients with mild delayed and nonunion fractures, while BMA combined with ACB potentially exhibits the lowest risk of adverse events. However, the quality of current evidence is low, and these findings require further validation through large-scale RCTs.

**Supplementary Information:**

The online version contains supplementary material available at 10.1186/s12891-026-09973-w.

## Introduction

Fracture nonunion is a pathological state where a fracture fails to heal within the expected time or cannot complete healing without clinical intervention [1]. The US FDA regards a fracture as nonunion if there is no evidence of healing for 3 consecutive months and healing has not been achieved for at least 9 months [2]. Fracture nonunion occurs in 2%–10% of fractures in high-income countries, and the rate may be higher in low-income countries [3]. The treatment cost per case of fracture nonunion reaches as high as 16,000 British pounds in the UK, 25,556 US dollars in the US and 4,788 Australian dollars in Australia respectively [4, 5]. Beyond the economic burden, fracture nonunion inflicts a dual physical and psychological blow on patients, among which the impact of long bone nonunion on physical health even outweighs that of chronic diseases such as congestive heart failure [6].

Autologous cancellous bone (ACB) graft is the current “gold standard” for treating delayed or nonunion fractures. It can fill bony defects and provide potential biological effects at the same time, yet it has certain limitations including limited bone material and insufficient osteogenic activity of the host [7]. Against this backdrop, biologics and physical therapies have gradually emerged with notable strengths including enhancing osteogenic activity, improving blood supply, regulating immunity and optimizing the mechanical microenvironment. They exhibit unique value in improving the healing rate of patients with fracture nonunion [8, 9].

A traditional meta-analysis reveals that platelet-rich plasma (PRP) combined with ACB can accelerate the healing of long bone nonunion, in contrast to ACB alone [10]. Rutten et al.’s randomized controlled trial (RCT) indicates that the average fracture healing time of patients receiving low-intensity pulsed ultrasound (LIPUS) is reduced by 39.8 days [11]. While various biologics and physical therapies have achieved preliminary clinical efficacy to date, there remains a lack of systematic comparative studies. Therefore, this study aims to systematically compare healing rate, healing time, and adverse event incidence among various biologics and physical therapies for delayed or nonunion fractures, and provide an evidence-based reference for clinical decision-making.

## Methods

The study was prospectively registered with PROSPERO on 12 September 2025 (registration ID: CRD420251145922). Furthermore, this study adhered to PRISMA-NMA (supplementary file 3).

### Search strategy

We searched PubMed, Web of Science, Embase, and the Cochrane Library from inception to October 13, 2025, for RCTs on delayed or nonunion fractures. Two researchers independently searched qualified trials, and discrepancies were settled by a third researcher. The concise search strategy is “Fracture, Ununited OR Ununited Fracture OR Ununited Fractures OR Non-Union OR Delayed Union OR Mal-Union OR Nonunion OR Nonunions OR bone ununion”. (Search strategy in supplementary file 1)

### Inclusion and exclusion criteria

Inclusion criteria: the study type was RCT; patients diagnosed with delayed or nonunion fractures (no healing for ≥ 9 months with no healing signs for 3 consecutive months; delayed union is defined as no obvious healing within 4 months after fracture [12]); interventions consisted of biologics or physical therapies; controls involved other biologics or physical therapies excluding the trial intervention, ACB, blank controls, or placebo treatment without active ingredients; at least one pre-specified outcome should be reported.

Exclusion criteria: animal experiments; in vitro experiments or other non-clinical studies; studies failing to report prespecified outcome measures; studies with incomplete data that could not be obtained even after contacting the corresponding author, or with unavailable full texts; reviews, case reports, conference abstracts.

### Data extraction

The following procedures were carried out by two independent researchers. Any discrepancies arising were arbitrated by a third researcher who was also responsible for data verification. For the included studies, two independent researchers collected the following key information: lead author, publication year, sample size, mean age, fracture site, intervention measures, control measures and follow-up details.

Primary outcomes were healing rate, healing time and adverse events. For continuous variables (healing time), the mean and standard deviation of post-intervention healing time were extracted. For pooled analysis, the values of healing time with inconsistent units across the included studies were uniformly converted to weeks as the standard unit. For dichotomous variables (healing rate and adverse events), event counts and total participants in each study were extracted separately. For included multi-arm studies, this study adopted the recommended approach specified in Sect. 23.3.4 of the Cochrane Handbook, pooling all experimental arms with consistent intervention types within the same study into a single experimental arm. Of the included trials, only Angelo et al. [13] (both experimental arms received extracorporeal shockwave therapy [ESWT] intervention) and Bilic et al. [14] (both experimental arms received bone morphogenetic protein [BMP] combined with ACB) were three-arm studies. For these two studies, we first merged the two experimental intervention arms of the same type within each study into a single experimental arm, and then conducted comparative analyses between the merged arm and the control arm. This was intended to avoid the excessive inflation of the control arm’s weight in the pooled analysis caused by its duplicate inclusion.

### Quality assessment

We employed the Cochrane Risk of Bias tool for RCTs to assess the risk of bias in the included studies. The assessed items included: random sequence generation, allocation concealment, blinding, completeness of outcome data, and selective reporting of outcomes. This assessment was conducted by two reviewers; any discrepancies were resolved by a third reviewer.

### Data synthesis and analysis

All analyses used Stata 18.0, and network meta-analyses were conducted via the mvmeta command within a frequentist framework. Dichotomous variables (healing rate and adverse events) were quantified with odds ratio (OR) as the effect size measure. For healing rate, OR > 1 means better outcomes in the intervention group (with a higher healing rate); for adverse events, OR < 1 means better outcomes in the intervention group (with a lower risk of adverse events). Due to inconsistent healing evaluation criteria (imaging/clinical) across studies, continuous variables (healing time) were quantified with standardized mean difference (SMD) as the effect size measure. SMD < 0 means better outcomes in the intervention group, with a shorter healing time. Given the existence of clinical and methodological heterogeneity across the included studies (e.g., uneven blinding), the random-effects model was adopted for all outcomes, with 95% confidence intervals (CIs) reported for each.

Network plots were generated on the basis of the analytical results. Nodes in the network plots represented different interventions, lines between the nodes denoted direct comparisons, and the thickness of the lines indicated the number of studies with direct comparisons. Different interventions were ranked according to the surface under the cumulative ranking curve (SUCRA), with values 0–1. The larger the value, the higher the probability that the intervention acts as the optimal treatment strategy. Traditional meta-analyses for the included direct comparisons were conducted through RevMan 5.4, with the random-effects model consistently applied for all analyses. Adjusted funnel plots were utilized to assess the potential publication bias in the present study. Furthermore, subgroup analyses were performed based on the classification of long and short bones.

## Results

### Search results and literature characteristics

**Fig. 1:**
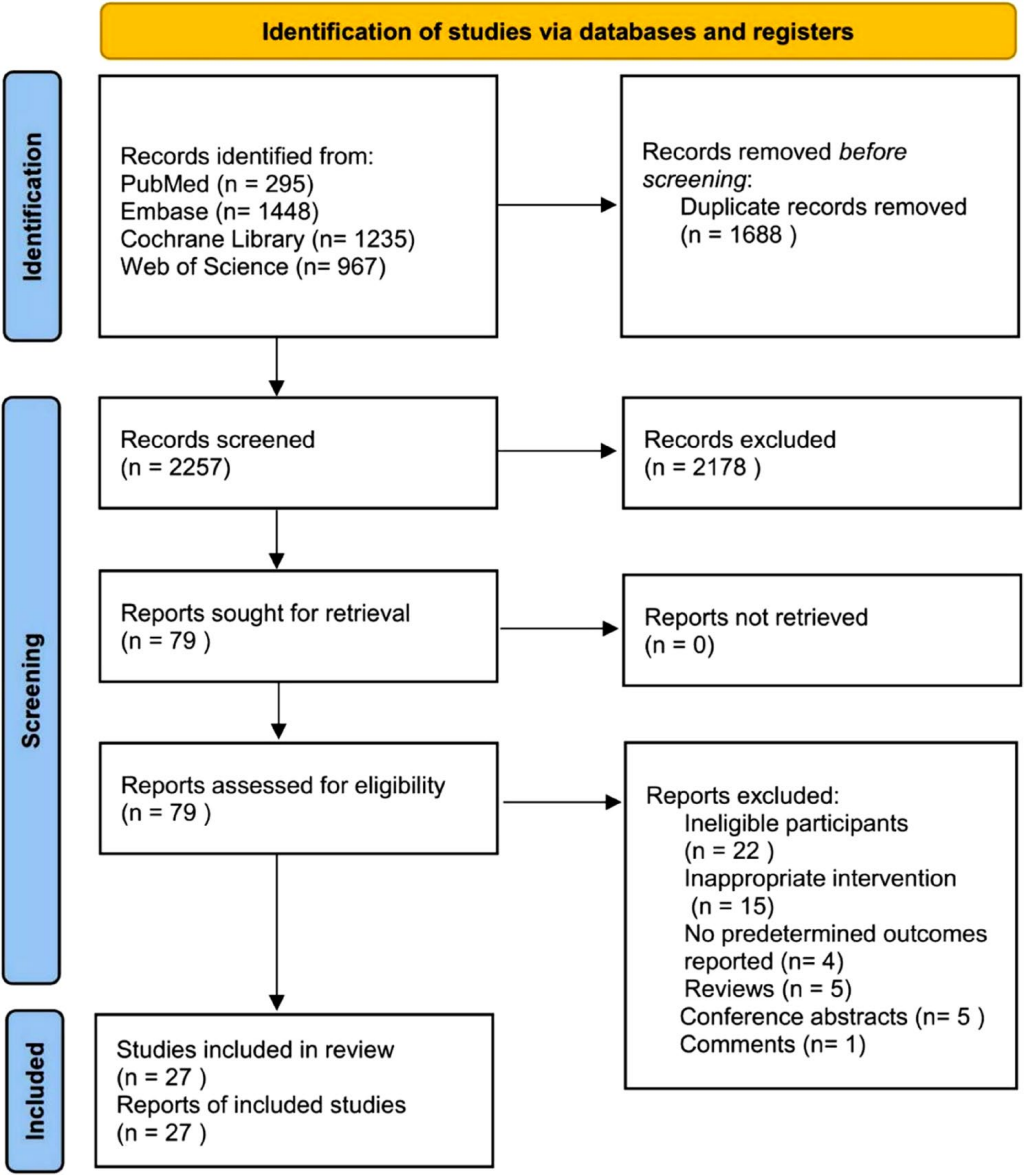
Literature search flow diagram

The initial review of the literature identified 3945 relevant references, with 118 full-text 149articles remaining after removing duplicate studies and conducting initial screening based on titles and abstracts. In line with our inclusion criteria, 27 RCTs involving 1495 participants were deemed eligible (Fig. 1). The included trials had sample sizes ranging from 8 to 142 participants. Post-treatment follow-up periods extended from 3 months to 7.6 years. Interventions were classified into two main categories: biological agents and physical therapies (Table 1). Physical therapies comprised LIPUS, ESWT and electromagnetic field (EMF); biologics included PRP, BMP, bone marrow aspirate (BMA, containing mesenchymal stem cells, MSCs), platelet-rich plasma concentrate (PRPc) and fibrin glue.

**Table 1:** Basic characteristics of the studies

Study	Sample	age	Fracture site	Interventions	Control	Follow-up
Neil 2024	142	27.1 ± 9.4 26.5 ± 12.1	Scaphoid	LIPUS	CONTROL	6.5 months
Shirin 2023	10	27.8 ± 5.54 25.6 ± 5.41	Scaphoid	BMA	ACB	3 months
Nicola 2022	66	41 ± 13 46 ± 12	Femur, Tibia	PRPc	PRP	12 months
Samuel 2018	40	34.3 ± 9.49 39.35 ± 10.39	Tibia	PRP	CONTROL	9 months
Philippe 2017	80	41.2 ± 18.2	Tibia	BMA + ACB	ACB	7.6 years
Carlos 2017	16	37.14 ± 10.22 38.88 ± 15.36	Humerus	PRP + ACB	ACB	9 months
Ghaffarpasand 2016	75	26.5 ± 5.8 26.3 ± 6.2	Femur, Tibia	PRP	CONTROL	12 months
Zhang 2016	24	33.45; 32.69	Tibia	BMA	CONTROL	12–34 months
Streit 2016	8	47 (24–63)	Fifth Metatarsal	EMF	CONTROL	6 months
Christian 2016	49	44 (19–77)	Ulnar, Radial	BMP + ACB	ACB	6 months
Zhai 2016	63	39.6 (23–50) 38.1 (20–49)	Femur, Tibia	BMA+ESWT	ESWT	12 months
Shi 2013	64	41.1 ± 14.5 38.4 ± 11.6	Femur, Tibia	EMF	CONTROL	3 months
Schofer 2010	101	42.6 ± 14.6 45.1 ± 11.9	Tibia	LIPUS	CONTROL	4 months
Angelo 2009	126	42.7 ± 5.9	Tibia, Femur	ESWT	CONTROL	6 months
Calori 2008	120	43 (19–65)	Humerus, Tibia	BMP	PRP	9 months
Ricardo 2006	21	26.7 (17–42)	Scaphoid	LIPUS	CONTROL	1–4 years
Calori 2006	29	47 ± 2.56 35.3 ± 1.76	Tibia Femur	BMP	PRP	9 months
Simonis 2002	34	32 (16–61)	Tibia	EMF	CONTROL	6 months
Friedlaender 2001	124	38 ± 16 34 ± 11	Tibia	BMP	ACB	24 months
Cook 1999	30	NR	Tibia	BMP	ACB	9 months
Scott 1994	21	43 (23–87)	Femur, Tibia	EMF	CONTROL	6 months
Sharrard 1990	45	34.7 (18–84) 45.4 (18–76)	Tibia	EMF	CONTROL	3 months
Barker 1984	16	34 (19–72)	Tibia	EMF	CONTROL	12 months
Meir 2013	24	38 (18–59) 43 (55 − 21)	Tibia	PRP + BMA	CONTROL	12 months
Bilic 2006	18	22 ± 5	Scaphoid	BMP + ACB	ACB	24 months
Yuan 2011	140	34 ± 2	Humerus, Tibia	BMA	ACB	3 months
Kunnasegaran 2024	9	41.7	Femur, Tibia	Fibrin + ACB	ACB	12 months

Data are presented as mean ± SD or mean (range)

*NR* No report, *BMA* Bone marrow aspirate, *ACB* Autologous cancellous bone, *PRP* Platelet-rich plasma, *BMP* Bone morphogenetic protein, *LIPUS* Low-intensity pulsed ultrasound, *ESWT* Extracorporeal shockwave therapy, *EMF* Electromagnetic field, *PRPc* Platelet-rich plasma concentrate

ACB, the well-recognized gold standard for nonunion treatment, was commonly administered in combination with the aforementioned single interventions in the incorporated clinical studies, thus forming the combined intervention groups. To assess the potential synergistic effect of combined interventions, the present study also included relevant studies focusing on such interventions. In summary, this study included 15 single and combined interventions: LIPUS, ESWT, EMF, PRP, BMP, BMA, PRPc, ACB, CONTROL, BMA + ACB, BMP + ACB, BMA+ESWT, PRP + BMA, PRP + ACB, and Fibrin + ACB.

### Literature quality assessment

**Fig. 2:**
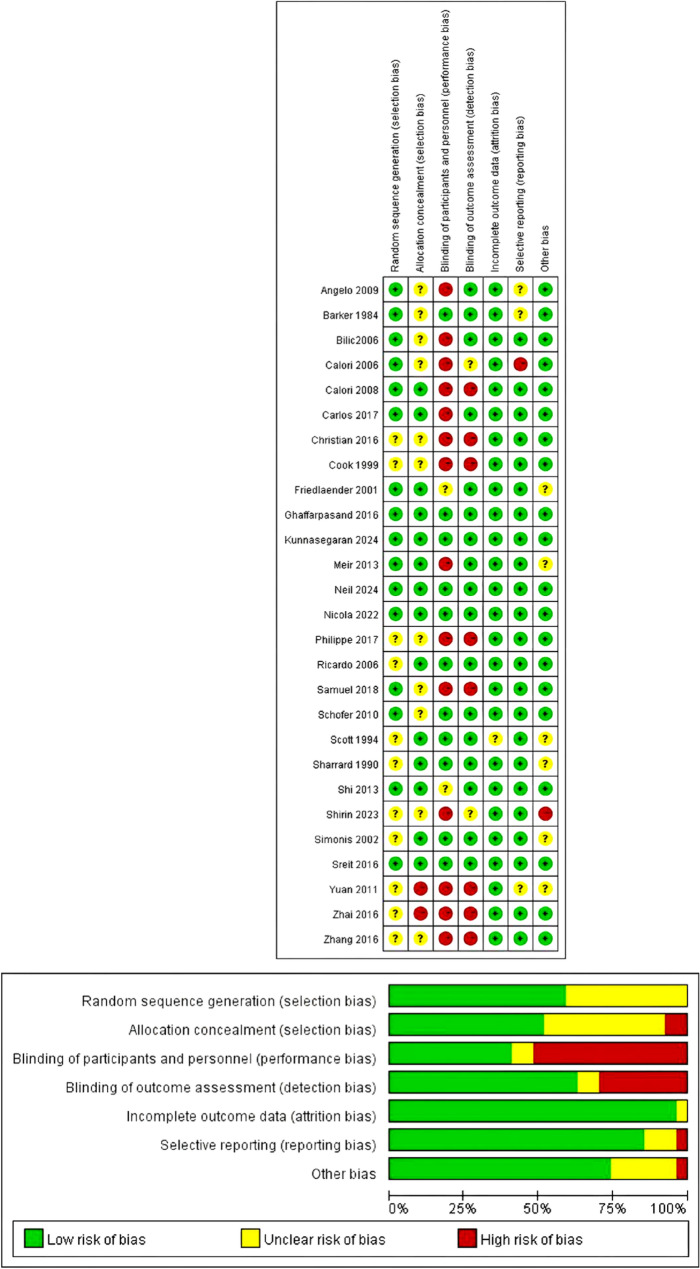
Risk of bias assessment chart

Figure 2 presents the risk of bias for each trial. Among the 27 trials, 16 studies (59%) exhibited a low risk of bias in random sequence generation; 15 studies (56%) had an unclear risk of bias for allocation concealment; 26 studies (96%) showed a low risk of bias related to incomplete outcome data; 23 studies (85%) were judged to be at low risk for selective reporting bias; 14 studies (52%) presented a low risk of bias in the domain of allocation concealment; only 11 studies (41%) performed blinding for study personnel and participants.

### Results of network meta-analysis

#### Healing rate

**Fig. 3:**
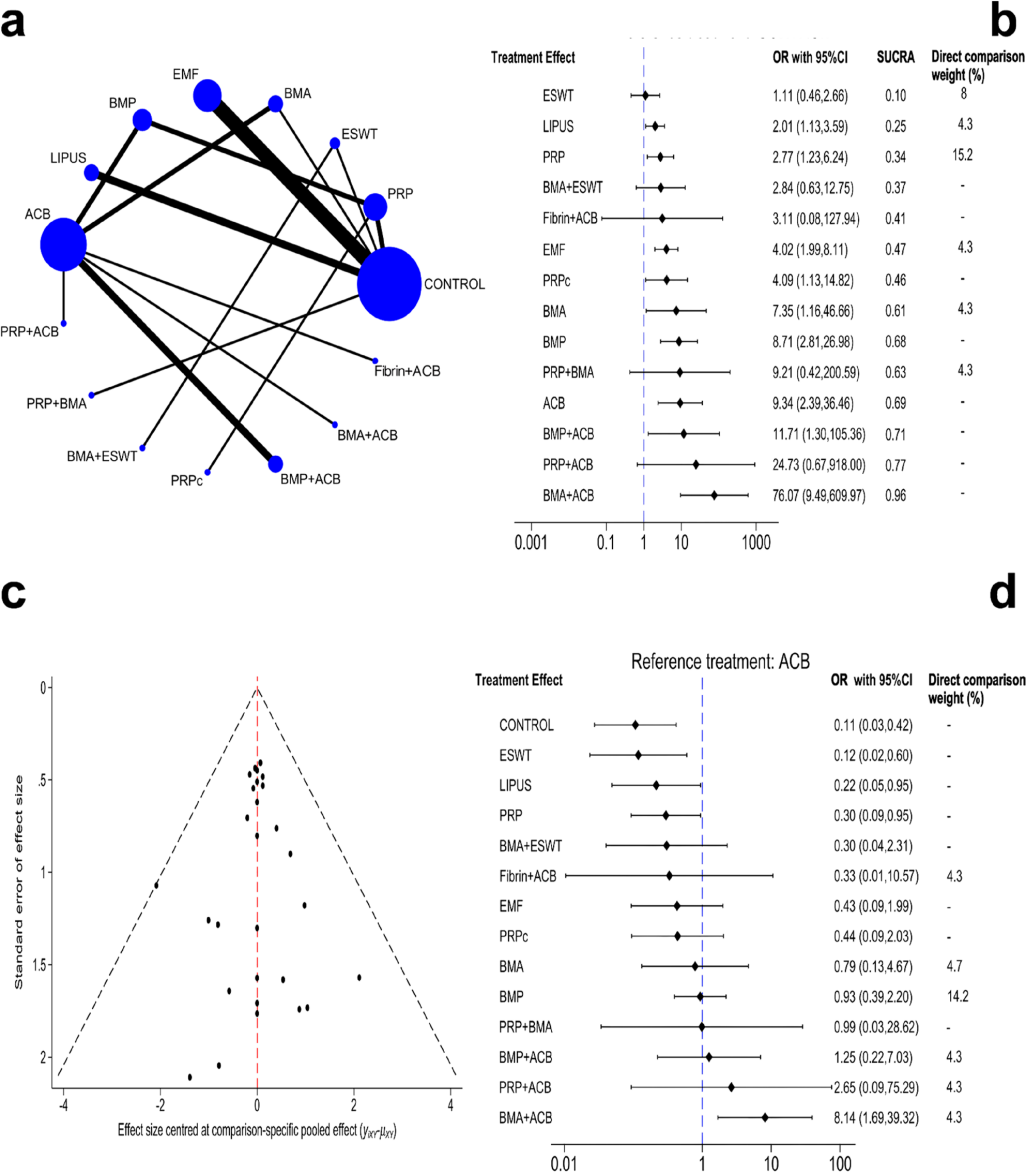
Healing rate analysis. Abbreviations for interventions are defined in the list of abbreviations. Effect sizes are expressed as ORs with 95% CIs; OR > 1 indicates higher healing rate with the intervention. **a** Network plot (Node size [proportional to sample size] and edge width [proportional to the number of studies]); (**b**) Forest plot comparing each intervention with control(“-” represents no direct comparison); (**c**) Funnel plot for healing rate; (**d**) Forest plot comparing each intervention with ACB

The present study included 27 RCTs [13–39], involving 1495 participants, and these trials encompassed 15 interventions including PRP, ESWT, BMA, EMF, BMP, LIPUS, ACB, PRPc, Fibrin glue, their combined interventions as well as the control group (Fig. 3a). Figure 3b demonstrates that the healing rate of most interventions was higher than that of the control group. Among these, BMA + ACB (OR = 76.07, 95% CI: 9.49 to 609.97) was found to potently promote the healing of fracture nonunion. Compared with ACB (Fig. 3d), only BMA + ACB (OR = 8.14, 95% CI: 1.69 to 39.32) achieved a higher healing rate. SUCRA ranking (Fig. 3b) revealed that BMA + ACB was the optimal intervention with the highest probability (96%), followed by PRP + ACB (77.3%) and BMP + ACB (70.5%). The cumulative probability plot is available in the supplementary file 1. The funnel plot did not detect publication bias (Fig. 3c).

#### Healing time

**Fig. 4:**
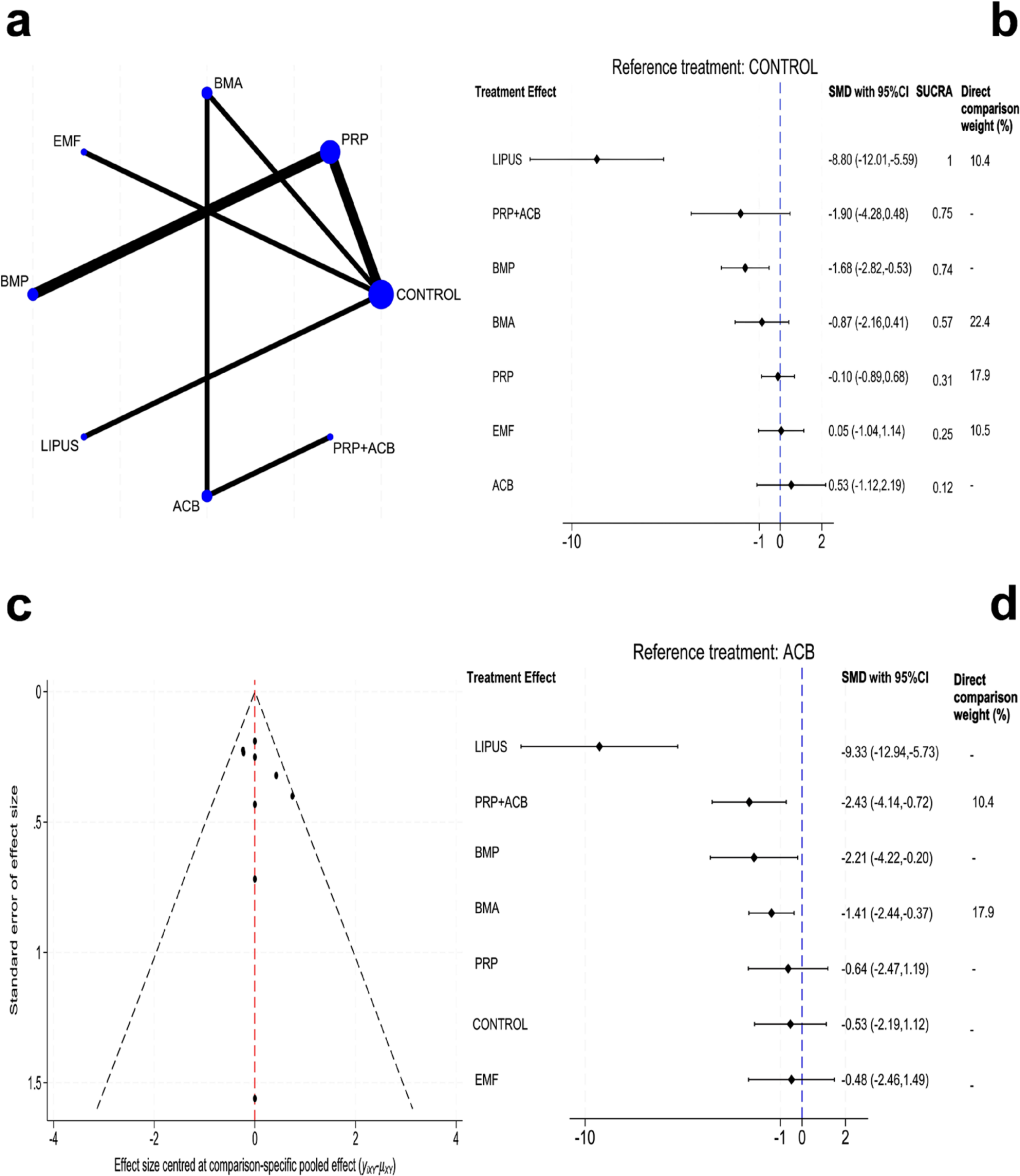
Healing time analysis. Abbreviations for interventions are defined in the list of abbreviations. Effect sizes are expressed as SMDs with 95% CIs; SMD < 0 indicates shorter healing time with the intervention. **a** Network plot; (**b**) Forest plot comparing each intervention with control; (**c**) Funnel plot for healing time; (**d**) Forest plot comparing each intervention with ACB

The present study enrolled 9 RCTs [17, 19, 21, 22, 26, 28–30, 35], involving 523 participants, covering 8 interventions: PRP, BMA, EMF, BMP, LIPUS, ACB, their combined interventions and the control group (Fig. 4a). Figure 4b demonstrates that only LIPUS (SMD = -8.8, 95% CI: -12.01 to -5.59) and BMP (SMD = -1.68, 95% CI: -2.82 to -0.53) were associated with significantly shorter healing time than the control group. Compared with ACB (Fig. 4d), LIPUS (SMD = -9.33, 95% CI: -12.94 to -5.73), PRP + ACB (SMD = -2.43, 95% CI: -4.14 to -0.72), BMP (SMD = -2.21, 95% CI: -4.22 to -0.20), and BMA (SMD = -1.41, 95% CI: -2.44 to -0.37) all significantly reduced healing time. SUCRA ranking revealed (Fig. 4b) that LIPUS had a 100% probability of being the optimal intervention for shortening healing time. The funnel plot failed to indicate obvious publication bias (Fig. 4c).

#### Adverse events

**Fig. 5:**
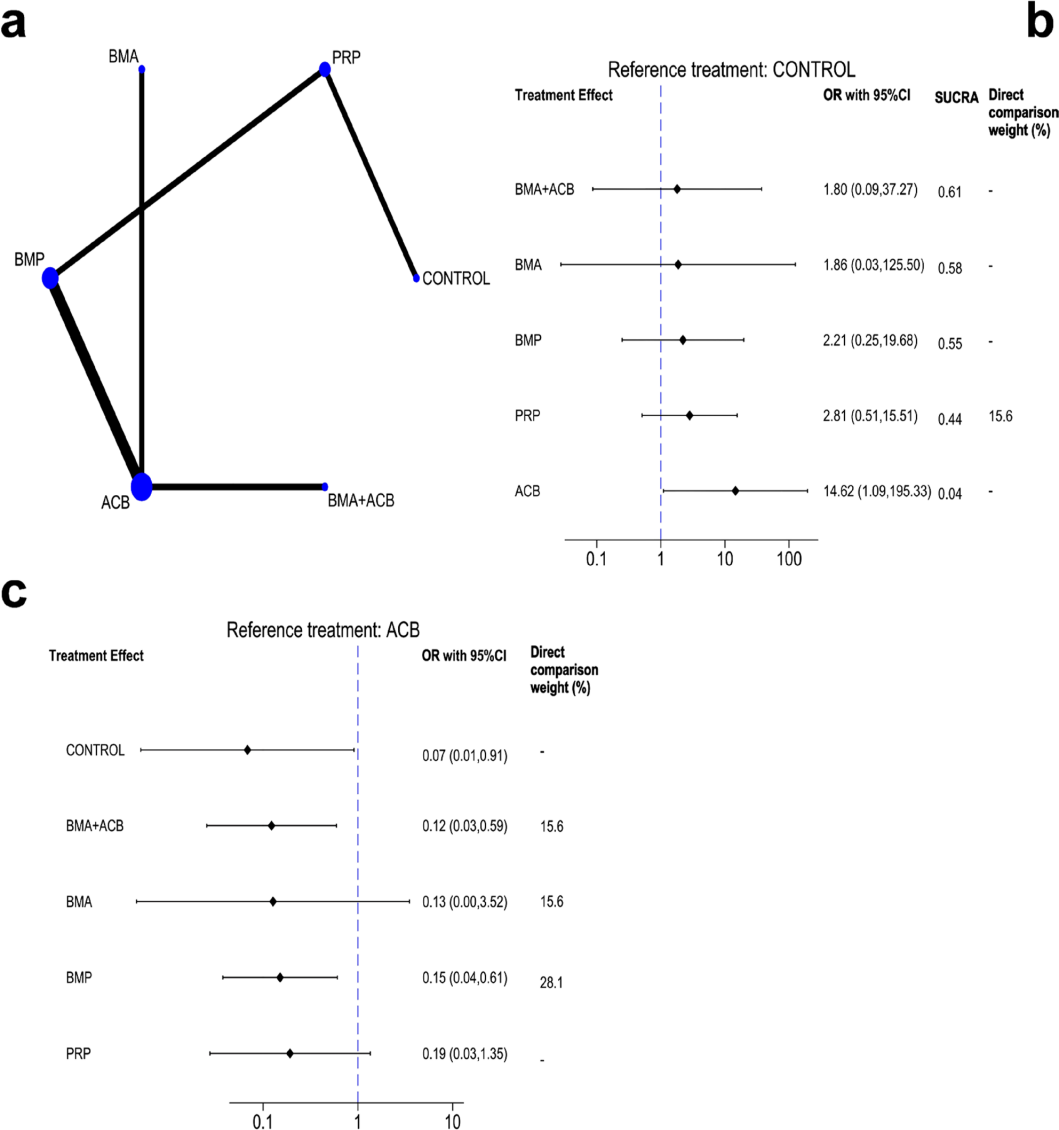
Adverse events analysis. Abbreviations for interventions are defined in the list of abbreviations. Effect sizes are expressed as ORs with 95% CIs; OR < 1 indicates lower risk of adverse events with the intervention. **a** Network plot; (**b**) Forest plot comparing each intervention with control; (**c**) Forest plot comparing each intervention with ACB

The present study incorporated 6 RCTs [18, 20, 21, 28, 32, 33], involving 437 participants, comprising 6 interventions: PRP, BMA, BMP, ACB, their combined interventions and the control group (Fig. 5a). Only ACB showed a higher incidence rate of adverse events versus the control group (OR = 14.62, 95% CI: 1.09 to 195.33) (Fig. 5b); the remaining interventions showed no significant difference in the incidence rate of adverse events from that of the control group. Compared with ACB (Fig. 5c), BMA + ACB (OR = 0.12, 95% CI: 0.03 to 0.59) and BMP (OR = 0.15, 95% CI: 0.04 to 0.61) exhibited a lower adverse event risk. SUCRA ranking indicated (Fig. 5b) that BMA + ACB (60.8%) emerged as the intervention with the optimal safety profile. Additionally, publication bias analysis was not performed owing to few included studies. As all participants for this outcome had long bone nonunion, no subgroup analysis was conducted. The adverse events reported in the present study included infection, donor site pain, hematoma (which resolved spontaneously thereafter), and transient numbness.

### Subgroup analysis

#### Healing rate

**Fig. 6:**
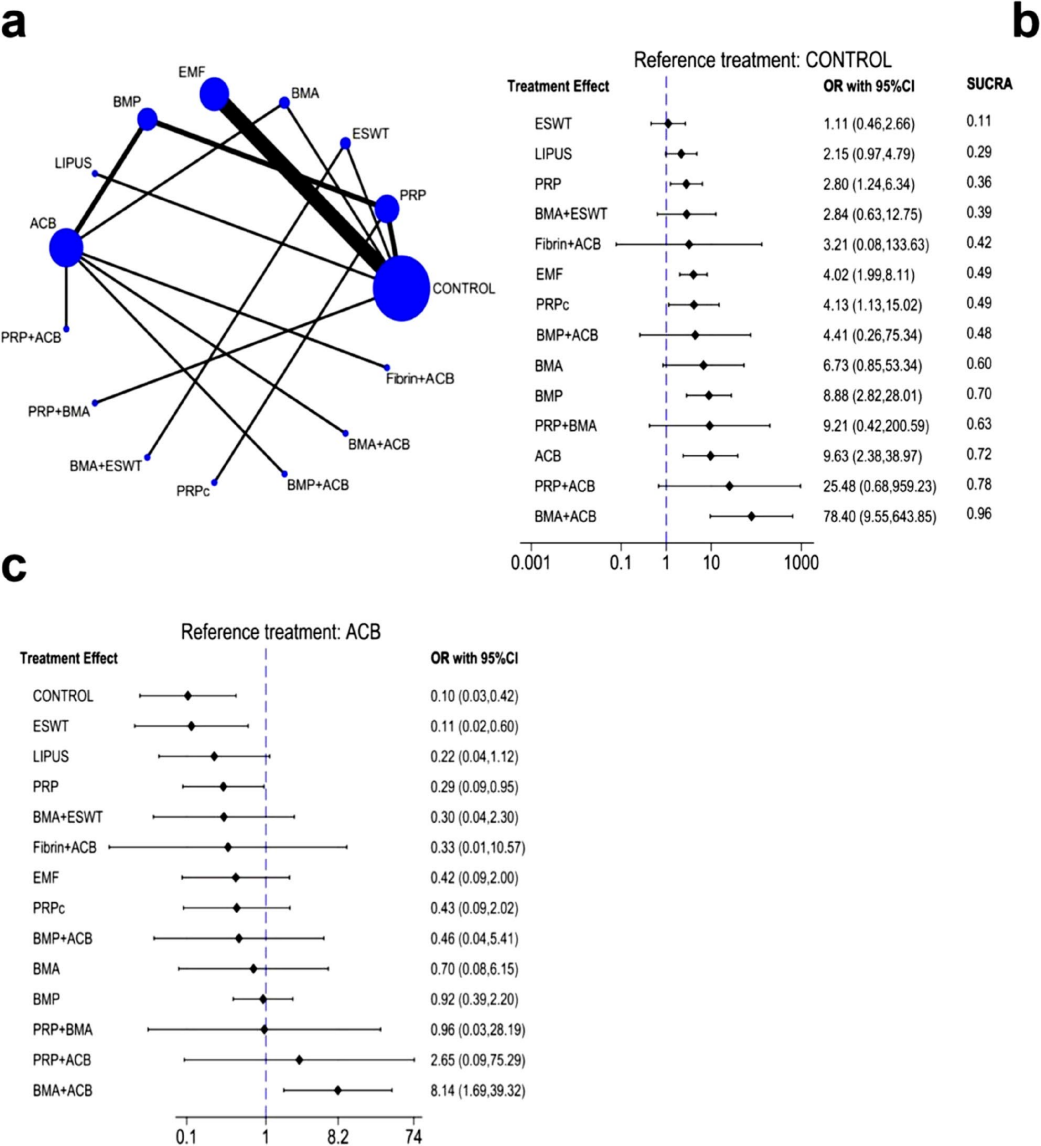
Healing rate subgroup analysis. Abbreviations for interventions are defined in the list of abbreviations. Effect sizes are expressed as ORs with 95% CIs; OR > 1 indicates higher healing rate with the intervention. **a** Network plot; (**b**) Forest plot comparing each intervention with control; (**c**) Forest plot comparing each intervention with ACB

The present study recruited 23 RCTs [13, 16–19, 21–28, 30–39], comprising 15 interventions (Fig. 6a). As shown in Fig. 6b, six interventions yielded a higher fracture healing rate than the control group: BMA + ACB (OR = 78.4, 95% CI: 9.55 to 643.85), ACB (OR = 9.63, 95% CI:2.38 to 38.97), BMP (OR = 8.88, 95% CI:2.82 to 28.01), PRPc (OR = 4.13, 95% CI:1.13 to 15.02), EMF (OR = 4.02, 95% CI:1.99 to 8.11) and PRP (OR = 2.8, 95% CI:1.24 to 6.34). Compared with ACB (Fig. 6c), only BMA + ACB (OR = 8.14, 95% CI: 1.69 to 39.32) exhibited a significantly higher fracture healing rate. SUCRA ranking (Fig. 6b) showed that BMA + ACB (96%) was the optimal intervention for long bone nonunion healing. Only 4 studies were included in the short bone subgroup, and the adopted interventions were all distinct; thus, no further network meta-analysis was conducted.

#### Healing time

**Fig. 7:**
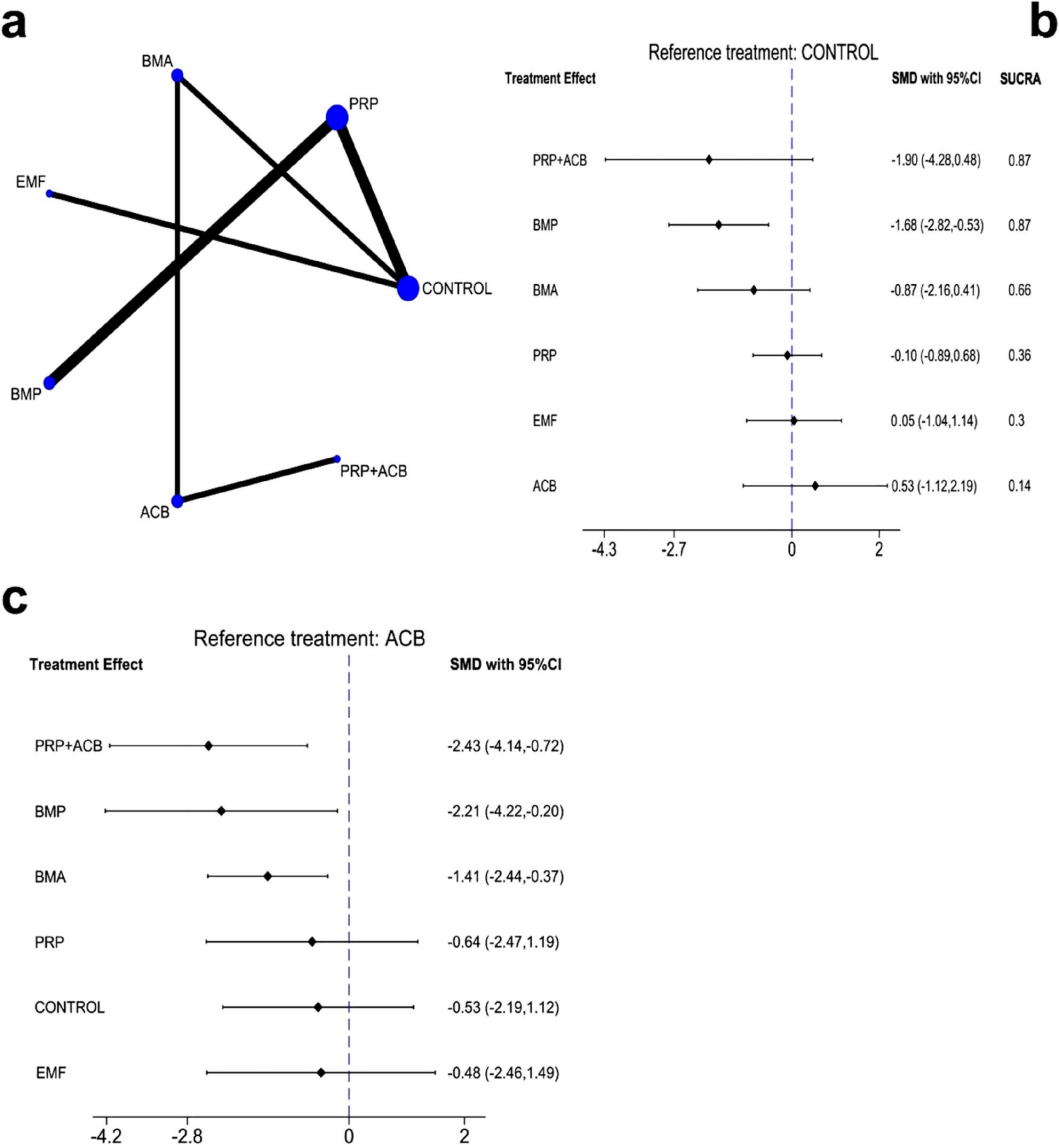
Healing time subgroup analysis. Abbreviations for interventions are defined in the list of abbreviations. Effect sizes are expressed as SMDs with 95% CIs; SMD < 0 indicates shorter healing time with the intervention. **a** Network plot; (**b**) Forest plot comparing each intervention with control; (**c**) Forest plot comparing each intervention with ACB

The present study enrolled 8 RCTs [17, 19, 21, 22, 26, 28, 30, 35], encompassing 7 interventions (Fig. 7a). Figure 7b demonstrates that only BMP significantly shortened healing time compared with the control group. Compared with ACB (Fig. 7c), PRP + ACB (SMD = -2.43, 95% CI: -4.14 to -0.72), BMP (SMD = -2.21, 95% CI: -4.22 to -0.20) and BMA (SMD = -1.41, 95% CI: -2.44 to -0.37) all exhibited a significantly reduced healing time. SUCRA ranking indicated (Fig. 7b) that BMP (87%) was the optimal intervention for shortening healing time in long bone nonunion. Only one study was available for the short bone subgroup, which precluded its inclusion in the analysis, and this study reported complete healing in all enrolled patients.

### Meta-analysis of direct comparisons

The network structural characteristics of the three outcomes in the present study showed consistency: the overall structure was connected, yet all were star-shaped (i.e., each intervention was directly connected to either the control group or ACB, while direct comparisons were absent between some interventions, which failed to form closed loops). Therefore, as no closed loops were present to compare direct and indirect evidence, the inconsistency test was not conducted. As a supplement, conventional meta-analyses were performed for direct comparisons with at least two studies per outcome.

#### Healing rate

For healing rate, a total of six pairs of direct comparisons, each with at least two studies included, were incorporated into the conventional meta-analysis (Table 2). Results revealed that versus the control group, PRP (OR = 3.09, *P* = 0.008), EMF (OR = 4.28, *P* = 0.003) and LIPUS (OR = 2.05, *P* = 0.02) all achieved a higher healing rate with low heterogeneity (I²<50%).

**Table 2:** Results of Direct Comparisons for Healing Rate

Comparison	Number of Studies	OR	95% CI	*P* value	I²(%)
PRP vs. CONTROL	2	3.09	1.34,7.10	0.008	0
EMF vs. CONTROL	6	4.28	1.61,11.33	0.003	38
LIPUS vs. CONTROL	3	2.05	1.14,3.67	0.02	0
BMA vs. ACB	2	1.25	0.22,7.03	0.8	0
BMP vs. ACB	2	0.83	0.34,2.00	0.67	0
BMP + ACB vs. ACB	2	0.93	0.13,6.85	0.94	0

#### Healing time

**Fig. 8:**
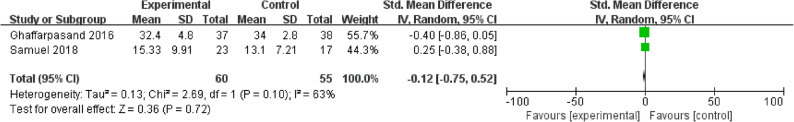
Forest plot: PRP vs. CONTROL for healing time

**Fig. 9:**
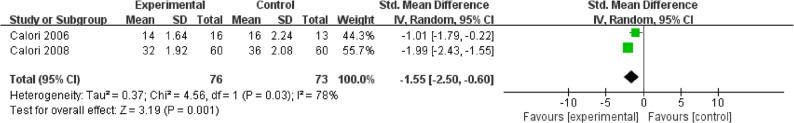
Forest plot: BMP vs. PRP for healing time

For healing time, only two direct comparisons (PRP vs. CONTROL and BMP vs. PRP) met the criteria for conventional meta-analysis, each with two studies. All other direct comparisons were based solely on a single study and were not subjected to pooled analysis. PRP and control showed no significant difference in healing time (Fig. 8) (SMD = -0.12, 95% CI: -0.75 to 0.52, *P* = 0.72). Compared with PRP, BMP significantly decreased the healing time (Fig. 9) (SMD = -1.55, 95% CI: -2.50 to -0.60, *P* = 0.001, I² = 78%). However, this comparison presented high heterogeneity. As only two studies were included, the sources of heterogeneity could not be investigated through subgroup analysis or sensitivity analysis. We speculate that the heterogeneity may result from inconsistent healing criteria used in the two studies. One adopted clinical healing criteria [30], while the other adopted imaging healing criteria [28].

#### Adverse events

**Fig. 10:**
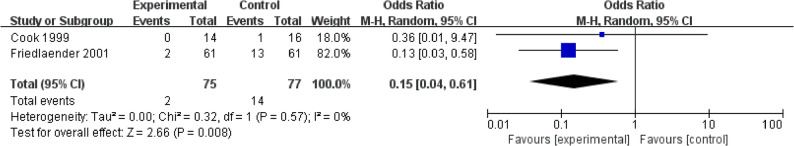
Forest plot: BMP vs. ACB for adverse events

For adverse events, only the direct comparison between BMP and ACB included two studies. All other direct comparisons were based solely on a single study, and no pooled analysis was performed for these comparisons. The results revealed that BMP resulted in a lower incidence of adverse events compared with ACB (OR = 0.15, 95% CI: 0.04 to 0.61, *P* = 0.008, I²=0), indicating good consistency across studies (Fig. 10).

### Quality of evidence

The quality of evidence for all outcomes was low, and was downgraded mainly due to imprecision, publication bias, and other factors. Detailed assessments are available in supplementary file 2.

## Discussion

This study conducted a systematic comparison of the clinical efficacy of various biologics and physical therapies for delayed and nonunion fractures. For fracture healing rate, BMA + ACB yielded a significant improvement; furthermore, the results of the long bone subgroup analysis were consistent with the overall analysis. For healing time, LIPUS may significantly shorten the healing time, and BMP could notably shorten it in the long bone subgroup. For adverse events, BMA + ACB may present the lowest risk of adverse events occurrence. This study also found that BMP may be significantly superior to ACB in both shortening healing time and reducing the incidence of adverse events. While biologics and physical therapies have been initially applied in clinical practice and demonstrated certain therapeutic efficacy, high-quality RCTs on them remain relatively scarce. In this study, comparisons of some interventions were based only on single or a small number of studies, leading to wide confidence intervals for effect size estimation. Caution is needed when interpreting the results. Despite these limitations, our study first and comprehensively analyzed various biologics and physical therapies for delayed or nonunion fractures. It provides clinicians with a reference for individualizing treatment plans.

At present, ACB represents the gold standard for managing nonunion fractures, for its ability to combine osteogenicity, osteoinductivity, and osteoconductivity [40]. However, due to limitations such as the limited volume of harvestable bone and decreased graft viability with age, its clinical efficacy varies among individuals, and some patients may even require secondary surgery [41]. This study found that BMA combined with ACB not only yielded the highest fracture healing rate but also was associated with the lowest incidence of adverse events. Yang et al. drew the same conclusion [42]. Both BMA and ACB are harvested from the iliac crest, yet studies have confirmed that BMA has a lower donor site morbidity [43]. BMA is not only rich in MSCs capable of direct osteogenic differentiation but also possesses strong osteoinductive capacity. Moreover, it secretes various growth factors and exosomes to regulate the fracture healing microenvironment, thus compensating for ACB’s deficiencies in the quantity of MSCs and the concentration of growth factors [41, 44, 45].

Furthermore, we found that compared with the control group, PRP + ACB had the second highest fracture healing rate, after BMA + ACB. Unlike MSCs-rich BMA, PRP is a platelet concentrate, and its therapeutic advantage lies in its ability to release multiple growth factors at high concentrations. Basic research has demonstrated that PDGF released by PRP stimulates osteoblast proliferation, VEGF enhances local angiogenesis, and TGF-β drives the differentiation of MSCs into osteoblasts; these factors jointly participate in the key processes of callus formation and bone matrix synthesis [46–48]. Traditional meta-analyses have confirmed that PRP monotherapy can serve as an alternative option for nonunion fractures [49]. This study not only validated this conclusion, but also demonstrated that PRP + ACB achieved a significantly higher fracture healing rate than PRP alone. This may be because PRP can release abundant growth factors but cannot directly provide the MSCs present in ACB and BMA; these cells can differentiate directly into osteoblasts and act as the core cells that drive fracture healing [50].

Among biologics for treating fracture nonunions, BMP is the most widely used in clinical practice. It is also the only growth factor proven to induce the differentiation of osteogenic progenitor cells into both chondrogenic and osteoblastic precursor cells in vivo [51]. De Long and Dinopulos noted that unlike other biologics, only BMP has obtained Level 1 clinical evidence to date, which can be clearly used as an alternative to ACB [52, 53]. This study found that BMP significantly shortens fracture healing time and reduces adverse events compared with ACB, consistent with Yang et al.‘s network meta-analysis [42]. However, the clinical application of BMP remains highly controversial [54], primarily owing to the risk of serious adverse events including heterotopic ossification, nerve injury, and osteolysis [55]. This is largely attributed to the lack of a precisely controllable delivery system, limiting its clinical utility [56]. In this study, BMP-related adverse events were few and all were infections, with no severe complications observed. Yet this may be related to sample size or dosage control. Thus, we still recommend that the dosage be carefully managed in clinical practice and that its delivery strategy be further optimized.

As a well-established non-invasive technique, LIPUS possesses unique clinical value. It promotes bone tissue regeneration by stimulating cellular activity at the fracture site with ultrasound waves, which is specifically reflected in enhancing the proliferation, differentiation and migration of periosteal cells and MSCs toward the fracture lesion [57, 58]. Multiple studies have confirmed that LIPUS accelerates fresh fracture healing and enhances nonunion recovery rates [59, 60]. This study further identified that LIPUS may significantly shorten the healing time in such patients. As a non-invasive therapy, LIPUS provides a vital alternative for patients who cannot tolerate surgical intervention. Furthermore, a health economic evaluation by the UK’s National Institute for Health and Care Excellence has shown that LIPUS, as a surgical alternative for fracture nonunion, can save approximately $1,726 per patient in medical costs [61].

However, an in-depth analysis of existing LIPUS-related clinical studies reveals that these studies rarely include complicated cases of delayed or nonunion fractures, making it difficult to reflect the actual clinical conditions of the nonunion patient population [7]. Therefore, LIPUS is more suitable for cases without surgical indications that are in stable condition and have partial potential for spontaneous healing; in clinical practice, the application of LIPUS for non-surgical treatment in such cases is more likely to achieve rapid healing of bone tissue.

Although BMA, PRP, BMP and LIPUS possess the above biological basis and provide a theoretical foundation for their clinical application in promoting bone healing and regulating the microenvironment, their mechanistic rationality cannot compensate for the obvious deficiencies in existing clinical evidence. First, some pairwise comparisons of interventions relied only on single or a small number of studies, which resulted in wide confidence intervals for effect sizes. Although subgroup analyses were conducted and the healing rate results were basically consistent with those of the main analysis, the study conclusions still require cautious interpretation. Second, the overall evidence network has a star-shaped structure and fails to form closed evidence loops, making it impossible to perform inconsistency tests to verify the consistency between direct and indirect evidence. To evaluate the robustness, we conducted a conventional meta-analysis for the eligible direct comparisons. The results showed no significant heterogeneity in most comparisons, suggesting good internal consistency within the direct evidence. In addition, although the available baseline characteristics (e.g., age) were not obviously imbalanced across comparison groups, we still cannot rule out potential differences in unmeasured effect modifiers. The transitivity assumption was not strictly verified. Therefore, the findings of this study should be considered exploratory. Future meta-analyses based on individual patient data are needed to provide more reliable evidence. Third, fewer than 10 studies were included for healing time. Although no obvious publication bias was identified, results should be interpreted with caution. Last, blinding was difficult to apply to treatment providers, study participants and outcome assessors in some studies, which might bring about subjective bias in the evaluation of fracture healing and thus lead to a positive tendency in the study results.

Given the limitations of this study, future research should focus on breakthroughs in the following aspects: well-designed and adequately sized multicenter RCTs are recommended to overcome the shortcomings of limited studies and small samples. Future studies should unify intervention dosage, treatment procedures and outcome assessment criteria and focus on observing possible adverse events so as to improve the accuracy of pooled effect size and enhance the clinical applicability and comparability of research results. Second, the follow-up period of patients should be prolonged to comprehensively evaluate the long-term impact of interventions on patients’ functional recovery. Finally, priority should be given to conducting direct comparative studies between different biological agents, as well as between biological agents and physical therapies, to clarify the advantages and disadvantages of various protocols and provide higher-quality evidence for subsequent research.

## Conclusion

Preliminary evidence indicates that compared with ACB alone, among the currently available biologics and physical therapies, BMA combined with ACB may be associated with a higher fracture healing rate; LIPUS may significantly shorten the healing time of delayed or nonunion fractures; BMA combined with ACB may have the lowest risk of adverse events. When making treatment decisions, clinicians should consider the aforementioned findings, patients’ individual conditions, accessibility of medical resources, and their own professional judgment. However, the quality of current evidence is low, and the results represent only exploratory findings; the findings still warrant cautious interpretation and require further validation through a large number of high-quality RCTs in the future.

## Supplementary Information

Supplementary Material 1.

Supplementary Material 2.

Supplementary Material 3.

## Data Availability

Data generated and analysed herein are contained in this article, including additional file.

## Abbreviations

SUCRA: Surface under the cumulative ranking curve
RCTs: Randomized controlled trials
OR: Odds ratio
SMD: Standardized mean difference
95% CI: 95% Confidence interval
BMA: Bone marrow aspirate
ACB: Autologous cancellous bone
PRP: Platelet-rich plasma
BMP: Bone morphogenetic protein
LIPUS: Low-intensity pulsed ultrasound
ESWT: Extracorporeal shockwave therapy
EMF: Electromagnetic field
PRPc: Platelet-rich plasma concentrate
MSCs: Mesenchymal stem cells
