# Supplementary material for: Efficacy and safety of biological agents and physical therapies for delayed union or nonunion of fractures: a network meta-analysis of randomized controlled trials
Source: BMC Musculoskelet Disord. 2026 May 28;27:637. doi: 10.1186/s12891-026-09973-w (PMC13422364; doi:10.1186/s12891-026-09973-w)
Supplement: Supplementary file 1 — Supplementary Material 1. [file 12891_2026_9973_MOESM1_ESM.docx]

**Efficacy and Safety of Biological Agents and Physical Therapies for Delayed Union or Nonunion of Fractures: A Network Meta-Analysis of Randomized Controlled Trials**

| ****Supplement**** | ****Page**** |
| --- | --- |
| ****Supplement 1. Detailed information for all search strategy**** | ****2-3**** |
| ****Supplement 2. League Table**** | ****4-5**** |
| ****Supplement 3. SUCRA Probability Ranking Plot**** | ****6-8**** |
| ****Supplement 4. Prediction Interval Plot**** | ****9-10**** |
| ****Supplement 5.** Results of Subgroup Analysis** | ****11-14**** |

****Supplement 1. Detailed information for all search strategy****

**1.1 Web of science 967**

Fracture, Ununited OR Ununited Fracture OR Ununited Fractures OR Non-Union OR Delayed Union OR Mal-Union OR Nonunion OR Nonunions OR bone ununion (Title) or Fracture, Ununited OR Ununited Fracture OR Ununited Fractures OR Non-Union OR Delayed Union OR Mal-Union OR Nonunion OR Nonunions OR bone ununion (Abstract) 20988

randomized controlled trial OR randomized OR placebo (Title) or randomized controlled trial OR randomized OR placebo (Abstract) 1142781

1AND2 967

**1.2 Embase 1448**

自由词：'delayed fracture healing' OR 'delayed fracture union' OR 'delayed union' OR 'fracture healing impairment' OR 'fracture malunion' OR 'fracture nonunion' OR 'fracture union delay' OR 'fractures, malunited' OR 'fractures, ununited' OR 'impaired fracture union' OR 'impeded fracture healing' OR 'malunion' OR 'malunited fractures' OR 'non-union' OR 'nonunion' OR 'nonunion (fracture)' OR 'nonunion fracture' OR 'ununited fracture' OR 'ununited fractures' OR 'impaired fracture healing'

主题词：impaired fracture healing

#3 AND #4 1448

#4 ('randomized controlled trial':ti,ab,kw OR randomized:ti,ab,kw OR placebo:ti,ab,kw) AND [embase]/lim 1161000

#3 #1 OR #2 34616

#2

(('delayed fracture healing':ti,ab,kw OR 'delayed fracture union':ti,ab,kw OR 'delayed union':ti,ab,kw OR 'fracture healing impairment':ti,ab,kw OR 'fracture malunion':ti,ab,kw OR 'fracture nonunion':ti,ab,kw OR 'fracture union delay':ti,ab,kw OR 'fractures, malunited':ti,ab,kw OR 'fractures, ununited':ti,ab,kw OR 'impaired fracture union':ti,ab,kw OR 'impeded fracture healing':ti,ab,kw OR 'malunion':ti,ab,kw OR 'malunited fractures':ti,ab,kw OR 'non-union':ti,ab,kw OR 'nonunion':ti,ab,kw OR nonunion:ti,ab,kw) AND fracture:ti,ab,kw OR 'nonunion fracture':ti,ab,kw OR 'ununited fracture':ti,ab,kw OR 'ununited fractures':ti,ab,kw OR 'impaired fracture healing':ti,ab,kw) AND [embase]/lim 14329

#1

('impaired fracture healing'/exp OR 'impaired fracture healing') AND [embase]/lim 30164

**1.3 Cochrane Library 1235**

#1 MeSH descriptor: [Fractures, Ununited] explode all trees 208

#2(Fracture, Ununited OR Ununited Fracture OR Ununited Fractures OR Non-Union OR Delayed Union OR Mal-Union OR Nonunion OR Nonunions OR bone ununion):ti,ab,kw 1639

#3 #1 OR #2 1671

#4 (randomized controlled trial):pt OR (randomized):ti,ab,kw OR (placebo):ti,ab,kw 1536975

#5 #3 AND #4 1259

Trials 1235

**1.4 Pubmed 295**

| **Search number** | **Query** | **Sort By** | **Filters** | **Search Details** | **Results** |
| --- | --- | --- | --- | --- | --- |
| 7 | ((randomized controlled trial[Publication Type]) AND (randomized[Title/Abstract] OR placebo[Title/Abstract])) AND ((("Fractures, Ununited"[Mesh]) OR (Fracture, Ununited[Title/Abstract] OR Ununited Fracture[Title/Abstract] OR Ununited Fractures[Title/Abstract])) OR (Non-Union[Title/Abstract] OR Delayed Union[Title/Abstract] OR Mal-Union[Title/Abstract] OR Nonunion[Title/Abstract] OR Nonunions[Title/Abstract] OR bone ununion[Title/Abstract])) |  |  | "randomized controlled trial"[Publication Type] AND ("randomized"[Title/Abstract] OR "placebo"[Title/Abstract]) AND ("fractures, ununited"[MeSH Terms] OR ("fracture ununited"[Title/Abstract] OR "ununited fracture"[Title/Abstract] OR "ununited fractures"[Title/Abstract]) OR ("Non-Union"[Title/Abstract] OR "delayed union"[Title/Abstract] OR "Mal-Union"[Title/Abstract] OR "Nonunion"[Title/Abstract] OR "Nonunions"[Title/Abstract] OR (("bone and bones"[MeSH Terms] OR ("bone"[All Fields] AND "bones"[All Fields]) OR "bone and bones"[All Fields] OR "bone"[All Fields]) AND "ununion"[Title/Abstract]))) | 295 |
| 6 | (("Fractures, Ununited"[Mesh]) OR (Fracture, Ununited[Title/Abstract] OR Ununited Fracture[Title/Abstract] OR Ununited Fractures[Title/Abstract])) OR (Non-Union[Title/Abstract] OR Delayed Union[Title/Abstract] OR Mal-Union[Title/Abstract] OR Nonunion[Title/Abstract] OR Nonunions[Title/Abstract] OR bone ununion[Title/Abstract]) |  |  | "fractures, ununited"[MeSH Terms] OR ("fracture ununited"[Title/Abstract] OR "ununited fracture"[Title/Abstract] OR "ununited fractures"[Title/Abstract]) OR ("Non-Union"[Title/Abstract] OR "delayed union"[Title/Abstract] OR "Mal-Union"[Title/Abstract] OR "Nonunion"[Title/Abstract] OR "Nonunions"[Title/Abstract] OR (("bone and bones"[MeSH Terms] OR ("bone"[All Fields] AND "bones"[All Fields]) OR "bone and bones"[All Fields] OR "bone"[All Fields]) AND "ununion"[Title/Abstract])) | 29,559 |
| 5 | (randomized controlled trial[Publication Type]) AND (randomized[Title/Abstract] OR placebo[Title/Abstract]) |  |  | "randomized controlled trial"[Publication Type] AND ("randomized"[Title/Abstract] OR "placebo"[Title/Abstract]) | 413,868 |
| 4 | Non-Union[Title/Abstract] OR Delayed Union[Title/Abstract] OR Mal-Union[Title/Abstract] OR Nonunion[Title/Abstract] OR Nonunions[Title/Abstract] OR bone ununion[Title/Abstract] |  |  | "Non-Union"[Title/Abstract] OR "delayed union"[Title/Abstract] OR "Mal-Union"[Title/Abstract] OR "Nonunion"[Title/Abstract] OR "Nonunions"[Title/Abstract] OR (("bone and bones"[MeSH Terms] OR ("bone"[All Fields] AND "bones"[All Fields]) OR "bone and bones"[All Fields] OR "bone"[All Fields]) AND "ununion"[Title/Abstract]) | 22,893 |
| 3 | Fracture, Ununited[Title/Abstract] OR Ununited Fracture[Title/Abstract] OR Ununited Fractures[Title/Abstract] |  |  | "fracture ununited"[Title/Abstract] OR "ununited fracture"[Title/Abstract] OR "ununited fractures"[Title/Abstract] | 459 |
| 1 | "Fractures, Ununited"[Mesh] | Most Recent |  | "fractures, ununited"[MeSH Terms] | 11,893 |

****Supplement 2. League Table****

（1）Healing rate league table

| **BMA** | **0.40 (0.12,1.32)** | **2.65 (0.42,16.82)** | **1.45 (0.50,4.23)** | **3.14 (1.36,7.22)** | **0.73 (0.27,1.96)** | **3.37 (1.05,10.76)** | **8.91 (0.26,308.40)** | **3.32 (0.14,80.31)** | **1.02 (0.19,5.64)** | **1.47 (0.54,4.01)** | **4.22 (0.53,33.75)** | **27.42 (3.87,194.06)** | **1.12 (0.03,43.06)** | **0.36 (0.16,0.81)** |
| --- | --- | --- | --- | --- | --- | --- | --- | --- | --- | --- | --- | --- | --- | --- |
| **2.51 (0.76,8.30)** | **EMF** | **6.64 (0.86,51.46)** | **3.64 (1.18,11.20)** | **7.88 (1.88,32.98)** | **1.82 (0.64,5.21)** | **8.45 (1.67,42.73)** | **22.36 (0.54,922.45)** | **8.33 (0.34,205.14)** | **2.57 (0.76,8.67)** | **3.70 (0.78,17.58)** | **10.59 (0.99,112.87)** | **68.79 (7.18,659.11)** | **2.82 (0.06,128.20)** | **0.90 (0.38,2.18)** |
| **0.38 (0.06,2.40)** | **0.15 (0.02,1.17)** | **LIPUS** | **0.55 (0.08,3.95)** | **1.19 (0.19,7.37)** | **0.27 (0.04,1.90)** | **1.27 (0.21,7.55)** | **3.37 (0.08,149.25)** | **1.25 (0.03,45.56)** | **0.39 (0.04,4.18)** | **0.56 (0.07,4.55)** | **1.59 (0.13,19.00)** | **10.35 (0.96,111.53)** | **0.42 (0.01,20.70)** | **0.14 (0.02,0.86)** |
| **0.69 (0.24,2.02)** | **0.27 (0.09,0.85)** | **1.83 (0.25,13.19)** | **ACB** | **2.17 (0.57,8.19)** | **0.50 (0.20,1.24)** | **2.32 (0.50,10.75)** | **6.15 (0.15,244.20)** | **2.29 (0.10,53.97)** | **0.71 (0.13,3.70)** | **1.02 (0.23,4.40)** | **2.91 (0.29,29.22)** | **18.92 (2.10,170.15)** | **0.77 (0.02,33.97)** | **0.25 (0.12,0.50)** |
| **0.32 (0.14,0.73)** | **0.13 (0.03,0.53)** | **0.84 (0.14,5.25)** | **0.46 (0.12,1.75)** | **BMP** | **0.23 (0.06,0.82)** | **1.07 (0.45,2.54)** | **2.84 (0.09,90.02)** | **1.06 (0.04,28.15)** | **0.33 (0.05,2.14)** | **0.47 (0.13,1.73)** | **1.34 (0.20,9.23)** | **8.73 (1.45,52.53)** | **0.36 (0.01,12.60)** | **0.11 (0.04,0.36)** |
| **1.38 (0.51,3.73)** | **0.55 (0.19,1.57)** | **3.65 (0.53,25.33)** | **2.00 (0.81,4.96)** | **4.33 (1.22,15.41)** | **PRP+ACB** | **4.64 (1.06,20.38)** | **12.29 (0.32,477.71)** | **4.58 (0.20,105.20)** | **1.41 (0.28,7.05)** | **2.03 (0.50,8.34)** | **5.82 (0.60,56.43)** | **37.81 (4.36,328.01)** | **1.55 (0.04,66.49)** | **0.50 (0.28,0.89)** |
| **0.30 (0.09,0.95)** | **0.12 (0.02,0.60)** | **0.79 (0.13,4.67)** | **0.43 (0.09,1.99)** | **0.93 (0.39,2.20)** | **0.22 (0.05,0.95)** | **PRP+BMA** | **2.65 (0.09,75.29)** | **0.99 (0.03,28.62)** | **0.30 (0.04,2.31)** | **0.44 (0.09,2.03)** | **1.25 (0.22,7.03)** | **8.14 (1.69,39.32)** | **0.33 (0.01,10.57)** | **0.11 (0.03,0.42)** |
| **0.11 (0.00,3.88)** | **0.04 (0.00,1.84)** | **0.30 (0.01,13.17)** | **0.16 (0.00,6.46)** | **0.35 (0.01,11.17)** | **0.08 (0.00,3.16)** | **0.38 (0.01,10.74)** | **BMA+ESWT** | **0.37 (0.00,43.01)** | **0.11 (0.00,5.76)** | **0.17 (0.00,6.57)** | **0.47 (0.01,20.46)** | **3.08 (0.08,124.38)** | **0.13 (0.00,15.49)** | **0.04 (0.00,1.50)** |
| **0.30 (0.01,7.29)** | **0.12 (0.00,2.96)** | **0.80 (0.02,28.99)** | **0.44 (0.02,10.29)** | **0.95 (0.04,25.18)** | **0.22 (0.01,5.02)** | **1.01 (0.03,29.45)** | **2.68 (0.02,310.08)** | **ESWT** | **0.31 (0.01,9.50)** | **0.44 (0.02,12.52)** | **1.27 (0.03,55.94)** | **8.26 (0.20,340.24)** | **0.34 (0.00,42.20)** | **0.11 (0.00,2.36)** |
| **0.98 (0.18,5.37)** | **0.39 (0.12,1.31)** | **2.58 (0.24,27.94)** | **1.41 (0.27,7.41)** | **3.06 (0.47,20.04)** | **0.71 (0.14,3.53)** | **3.29 (0.43,24.92)** | **8.70 (0.17,435.36)** | **3.24 (0.11,99.69)** | **PRPc** | **1.44 (0.20,10.38)** | **4.12 (0.29,58.90)** | **26.75 (2.06,348.15)** | **1.10 (0.02,60.22)** | **0.35 (0.08,1.58)** |
| **0.68 (0.25,1.84)** | **0.27 (0.06,1.29)** | **1.80 (0.22,14.70)** | **0.98 (0.23,4.26)** | **2.13 (0.58,7.83)** | **0.49 (0.12,2.02)** | **2.29 (0.49,10.59)** | **6.05 (0.15,240.35)** | **2.25 (0.08,63.53)** | **0.70 (0.10,5.02)** | **PRP** | **2.87 (0.29,28.77)** | **18.61 (2.07,167.54)** | **0.76 (0.02,33.43)** | **0.24 (0.07,0.89)** |
| **0.24 (0.03,1.89)** | **0.09 (0.01,1.01)** | **0.63 (0.05,7.48)** | **0.34 (0.03,3.44)** | **0.74 (0.11,5.10)** | **0.17 (0.02,1.66)** | **0.80 (0.14,4.47)** | **2.11 (0.05,91.17)** | **0.79 (0.02,34.58)** | **0.24 (0.02,3.47)** | **0.35 (0.03,3.50)** | **BMP+ACB** | **6.49 (0.63,67.05)** | **0.27 (0.01,12.65)** | **0.09 (0.01,0.77)** |
| **0.04 (0.01,0.26)** | **0.01 (0.00,0.14)** | **0.10 (0.01,1.04)** | **0.05 (0.01,0.48)** | **0.11 (0.02,0.69)** | **0.03 (0.00,0.23)** | **0.12 (0.03,0.59)** | **0.33 (0.01,13.14)** | **0.12 (0.00,4.99)** | **0.04 (0.00,0.49)** | **0.05 (0.01,0.48)** | **0.15 (0.01,1.59)** | **BMA+ACB** | **0.04 (0.00,1.83)** | **0.01 (0.00,0.11)** |
| **0.89 (0.02,34.18)** | **0.36 (0.01,16.16)** | **2.36 (0.05,115.24)** | **1.29 (0.03,56.64)** | **2.80 (0.08,98.60)** | **0.65 (0.02,27.76)** | **3.00 (0.09,95.17)** | **7.94 (0.06,976.98)** | **2.96 (0.02,369.13)** | **0.91 (0.02,50.21)** | **1.31 (0.03,57.62)** | **3.76 (0.08,179.06)** | **24.43 (0.55,1090.60)** | **fibrin+ACB** | **0.32 (0.01,13.19)** |
| **2.77 (1.23,6.24)** | **1.11 (0.46,2.66)** | **7.35 (1.16,46.66)** | **4.02 (1.99,8.11)** | **8.71 (2.81,26.98)** | **2.01 (1.13,3.59)** | **9.34 (2.39,36.46)** | **24.73 (0.67,918.00)** | **9.21 (0.42,200.59)** | **2.84 (0.63,12.75)** | **4.09 (1.13,14.82)** | **11.71 (1.30,105.36)** | **76.07 (9.49,609.97)** | **3.11 (0.08,127.94)** | **CONTROL** |

1. Healing time league table

| **PRP** | **-0.77**  **(-2.28,0.74)** | **0.15**  **(-1.19,1.49)** | **-1.57**  **(-2.40,-0.75)** | **-8.70**  **(-12.00,-5.39)** | **0.64**  **(-1.19,2.47)** | **-1.79**  **(-4.30,0.71)** | **0.10**  **(-0.68,0.89)** |
| --- | --- | --- | --- | --- | --- | --- | --- |
| **0.77 (-0.74,2.28)** | **BMA** | **0.92 (-0.76,2.61)** | **-0.80 (-2.53,0.92)** | **-7.93 (-11.38,-4.47)** | **1.41 (0.37,2.44)** | **-1.03 (-3.03,0.97)** | **0.87 (-0.41,2.16)** |
| **-0.15 (-1.49,1.19)** | **-0.92 (-2.61,0.76)** | **EMF** | **-1.73 (-3.30,-0.15)** | **-8.85 (-12.24,-5.46)** | **0.48 (-1.49,2.46)** | **-1.95 (-4.56,0.67)** | **-0.05 (-1.14,1.04)** |
| **1.57 (0.75,2.40)** | **0.80 (-0.92,2.53)** | **1.73 (0.15,3.30)** | **BMP** | **-7.12 (-10.53,-3.72)** | **2.21 (0.20,4.22)** | **-0.22 (-2.86,2.42)** | **1.68 (0.53,2.82)** |
| **8.70 (5.39,12.00)** | **7.93 (4.47,11.38)** | **8.85 (5.46,12.24)** | **7.12 (3.72,10.53)** | **LIPUS** | **9.33 (5.73,12.94)** | **6.90 (2.91,10.90)** | **8.80 (5.59,12.01)** |
| **-0.64 (-2.47,1.19)** | **-1.41 (-2.44,-0.37)** | **-0.48 (-2.46,1.49)** | **-2.21 (-4.22,-0.20)** | **-9.33 (-12.94,-5.73)** | **ACB** | **-2.43 (-4.14,-0.72)** | **-0.53 (-2.19,1.12)** |
| **1.79 (-0.71,4.30)** | **1.03 (-0.97,3.03)** | **1.95 (-0.67,4.56)** | **0.22 (-2.42,2.86)** | **-6.90 (-10.90,-2.91)** | **2.43 (0.72,4.14)** | **PRP+ACB** | **1.90 (-0.48,4.28)** |
| **-0.10 (-0.89,0.68)** | **-0.87 (-2.16,0.41)** | **0.05 (-1.04,1.14)** | **-1.68 (-2.82,-0.53)** | **-8.80 (-12.01,-5.59)** | **0.53 (-1.12,2.19)** | **-1.90 (-4.28,0.48)** | **CONTROL** |

1. Adverse events league table

| **PRP** | **0.66 (0.01,31.08)** | **0.79 (0.20,3.08)** | **5.20 (0.74,36.55)** | **0.64 (0.05,7.83)** | **0.36 (0.06,1.96)** |
| --- | --- | --- | --- | --- | --- |
| **1.51 (0.03,70.97)** | **BMA** | **1.19 (0.03,43.40)** | **7.86 (0.28,217.10)** | **0.96 (0.02,38.01)** | **0.54 (0.01,36.23)** |
| **1.27 (0.32,4.99)** | **0.84 (0.02,30.79)** | **BMP** | **6.62 (1.65,26.61)** | **0.81 (0.10,6.65)** | **0.45 (0.05,4.03)** |
| **0.19 (0.03,1.35)** | **0.13 (0.00,3.52)** | **0.15 (0.04,0.61)** | **ACB** | **0.12 (0.03,0.59)** | **0.07 (0.01,0.91)** |
| **1.57 (0.13,19.20)** | **1.04 (0.03,40.82)** | **1.23 (0.15,10.06)** | **8.14 (1.69,39.32)** | **BMA+ACB** | **0.56 (0.03,11.56)** |
| **2.81 (0.51,15.51)** | **1.86 (0.03,125.50)** | **2.21 (0.25,19.68)** | **14.62 (1.09,195.33)** | **1.80 (0.09,37.27)** | **CONTROL** |

****Supplement 3. SUCRA Probability Ranking Plot****

1. Probability ranking plot for healing rate


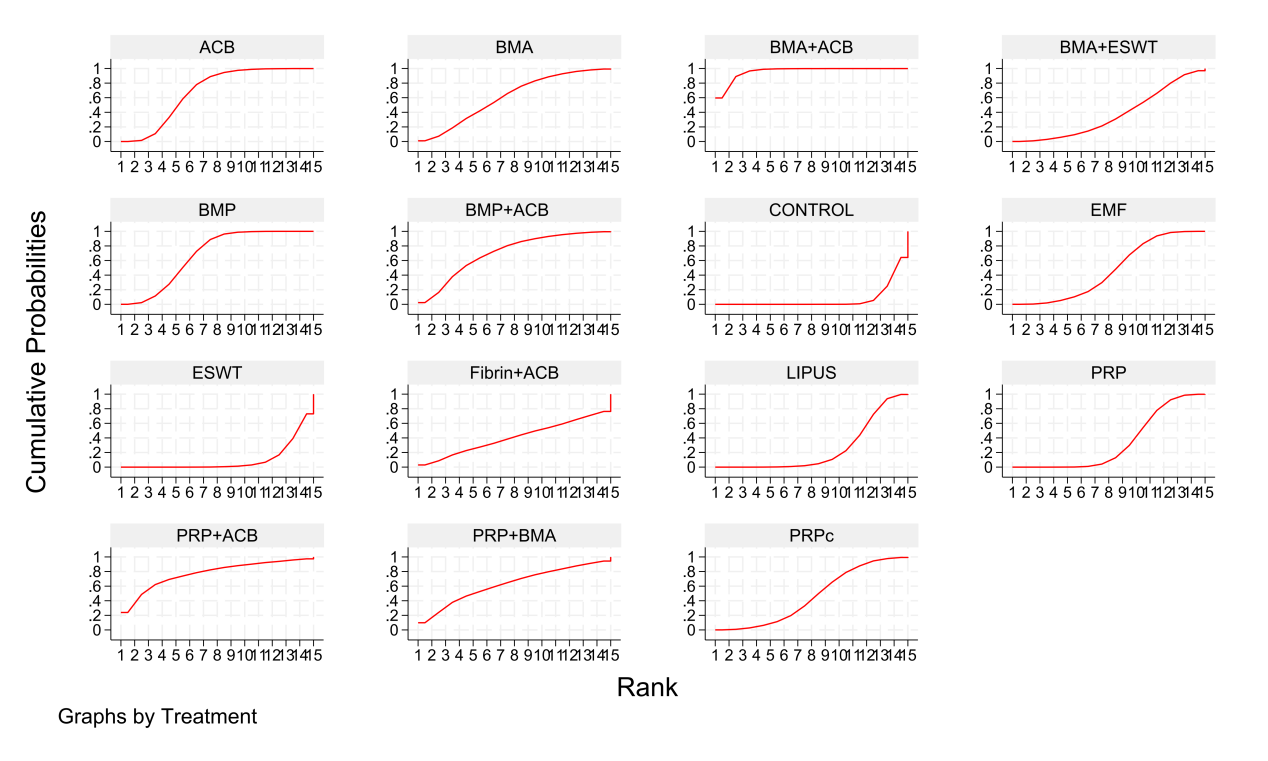


|Treatment | SUCRA | PrBest | MeanRank |

CONTROL | 6.8 | 0.0 | 14.0 |

PRP | 33.6 | 0.0 | 10.3 |

ESWT | 10.1 | 0.0 | 13.6 |

BMA | 61.0 | 1.0 | 6.5 |

EMF | 46.8 | 0.0 | 8.4 |

BMP | 67.7 | 0.0 | 5.5 |

LIPUS | 25.0 | 0.0 | 11.5 |

ACB | 68.6 | 0.0 | 5.4 |

PRP+ACB | 77.3 | 24.0 | 4.2 |

PRP+BMA | 62.7 | 9.9 | 6.2 |

BMA+ESWT | 36.9 | 0.1 | 9.8 |

PRPc | 46.3 | 0.1 | 8.5 |

BMP+ACB | 70.5 | 2.4 | 5.1 |

BMA+ACB | 96.0 | 59.7 | 1.6 |

Fibrin+ACB | 40.7 | 3.0 | 9.3 |

1. Probability ranking plot for healing time


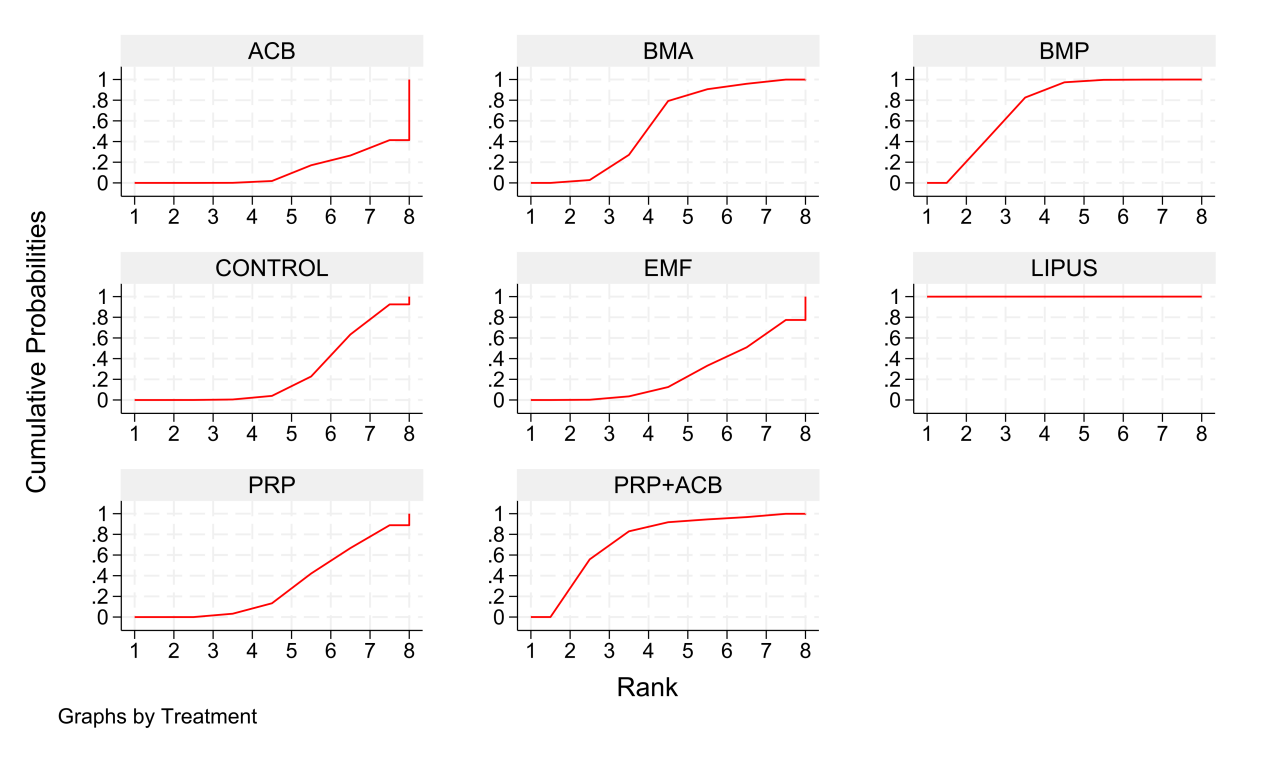


| Treatm~t | SUCRA | PrBest | MeanRank |

| CONTROL | 26.2 | 0.0 | 6.2 |

| PRP | 30.6 | 0.0 | 5.9 |

| BMA | 56.5 | 0.0 | 4.0 |

| EMF | 25.4 | 0.0 | 6.2 |

| BMP | 74.4 | 0.0 | 2.8 |

| LIPUS | 100.0 | 99.9 | 1.0 |

| ACB | 12.4 | 0.0 | 7.1 |

| PRP+ACB | 74.5 | 0.0 | 2.8 |

1. Probability ranking plot for adverse events


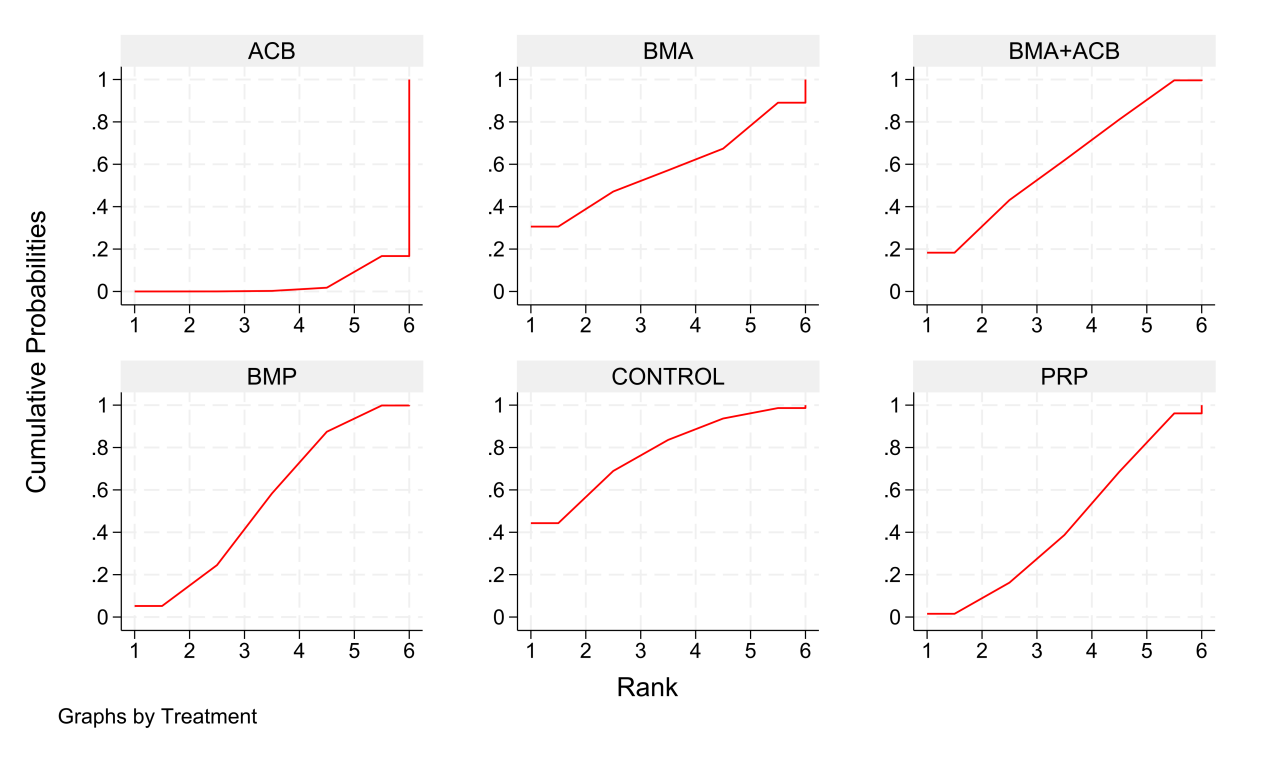


| Treatm~t | SUCRA | PrBest | MeanRank |

|----------+-------+--------+----------|

| CONTROL | 77.8 | 44.3 | 2.1 |

| PRP | 44.2 | 1.5 | 3.8 |

| BMA | 58.3 | 30.6 | 3.1 |

| BMP | 55.1 | 5.2 | 3.2 |

| ACB | 3.8 | 0.0 | 5.8 |

| BMA+ACB | 60.8 | 18.3 | 3.0 |

****Supplement 4. Prediction Interval Plot****

1. Prediction interval plot of healing rate

**
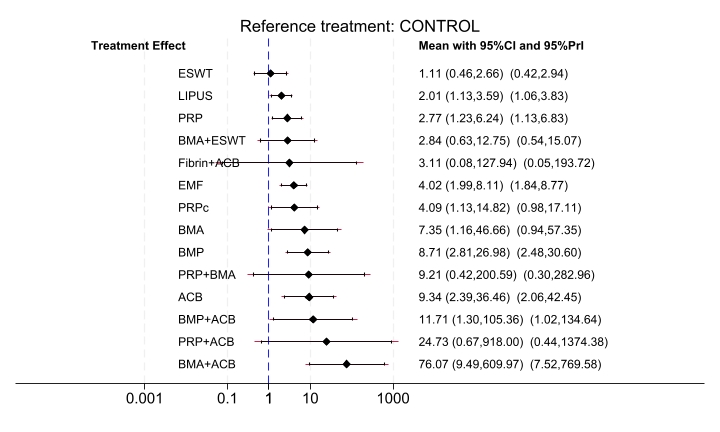
**

Fig.1 Prediction interval plot comparing each intervention with control

**
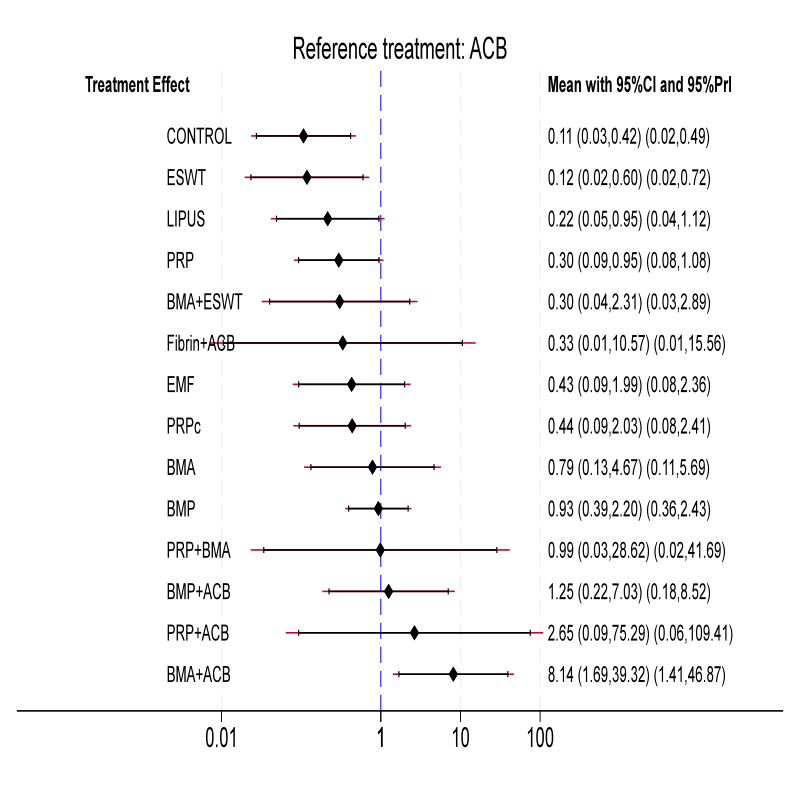
**

Fig.2 Prediction interval plot comparing each intervention with ACB

1. Prediction interval plot of healing time


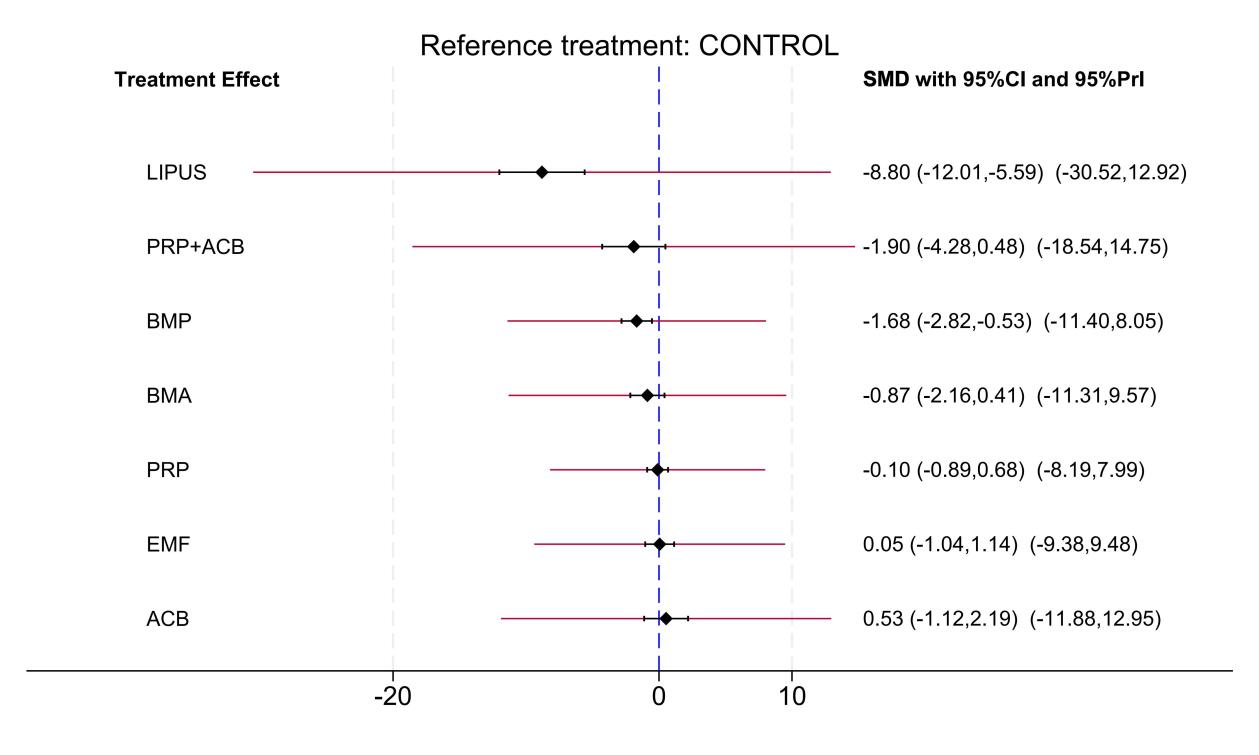


Fig.3 Prediction interval plot comparing each intervention with control

**
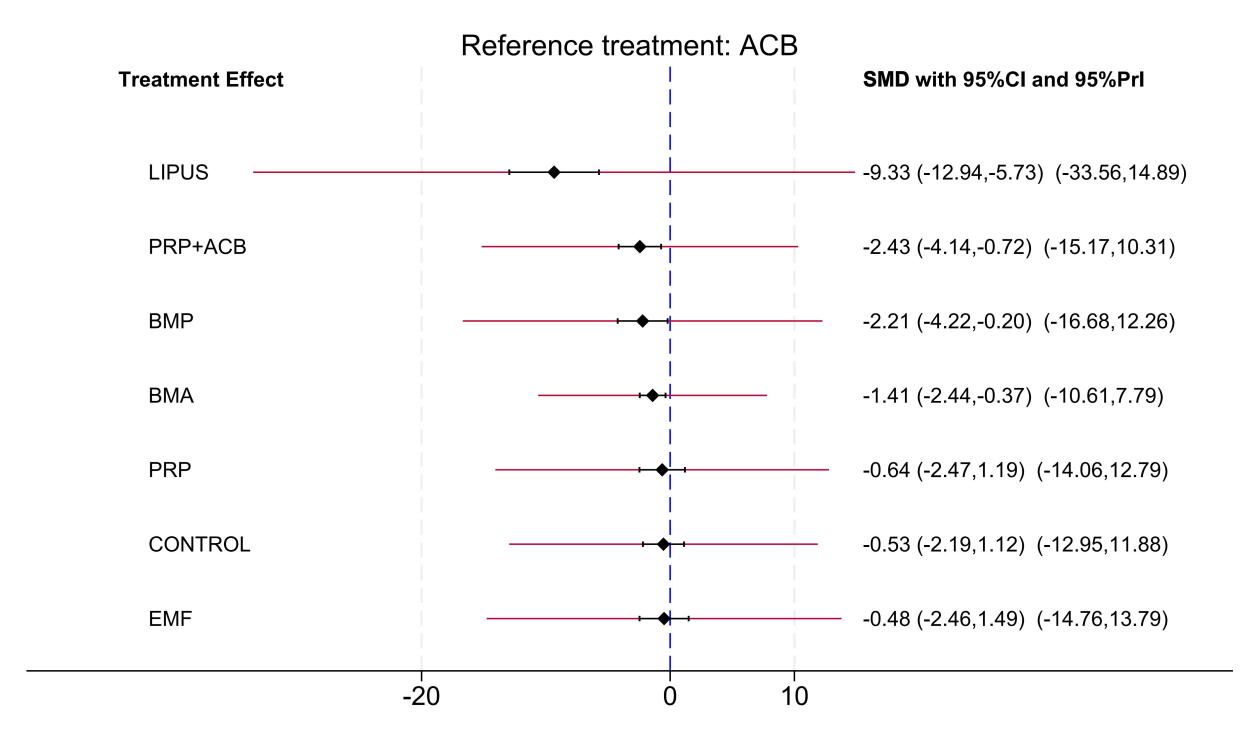
**

Fig.4 Prediction interval plot comparing each intervention with ACB

****Supplement 5.** Results of Subgroup Analysis**

**5.1 Results of the long bone subgroup for healing rate**


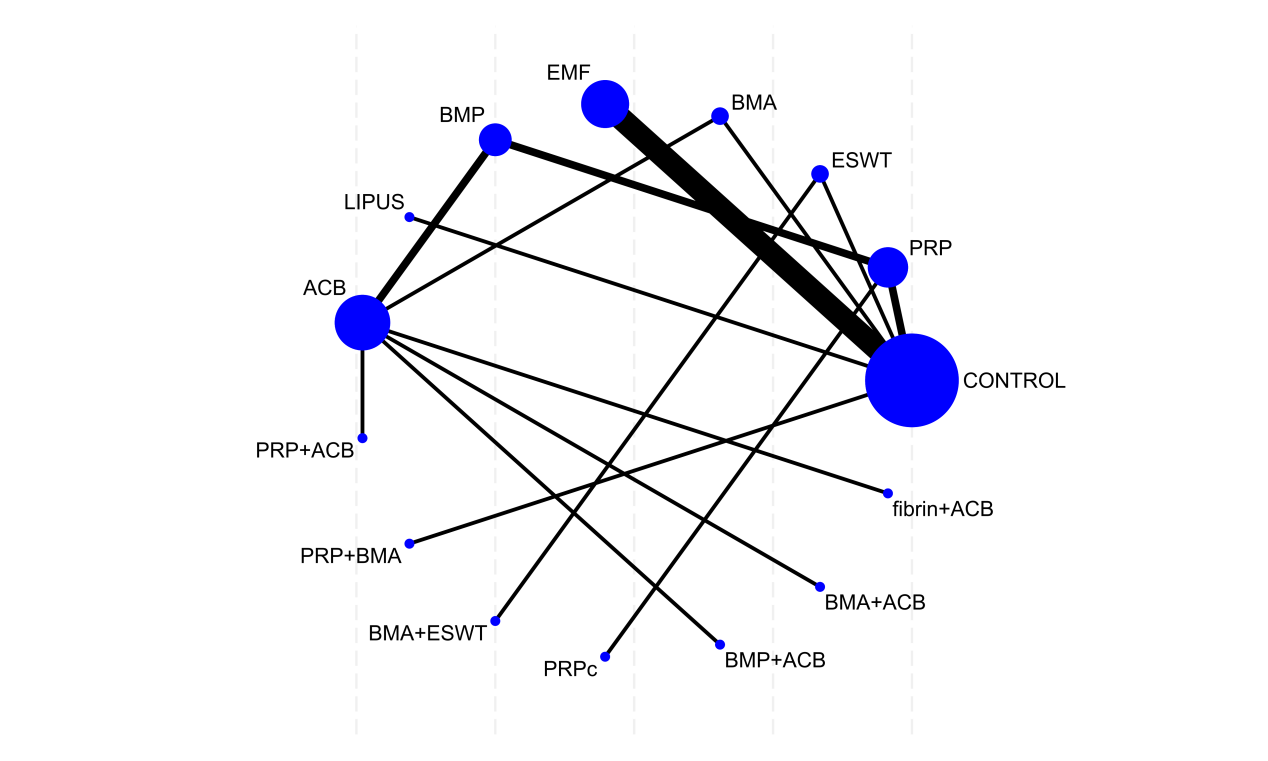


Fig.1 Network plot of results


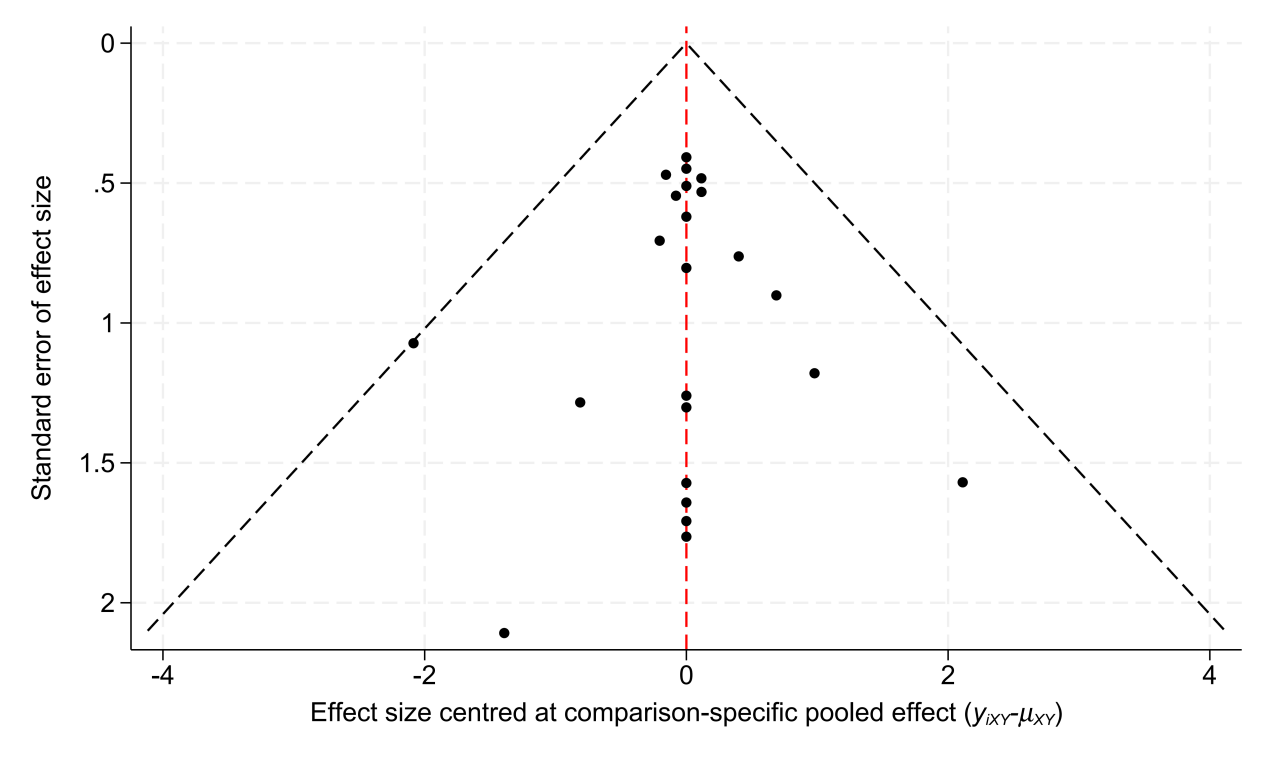


Fig.2 Funnel plot for healing rate


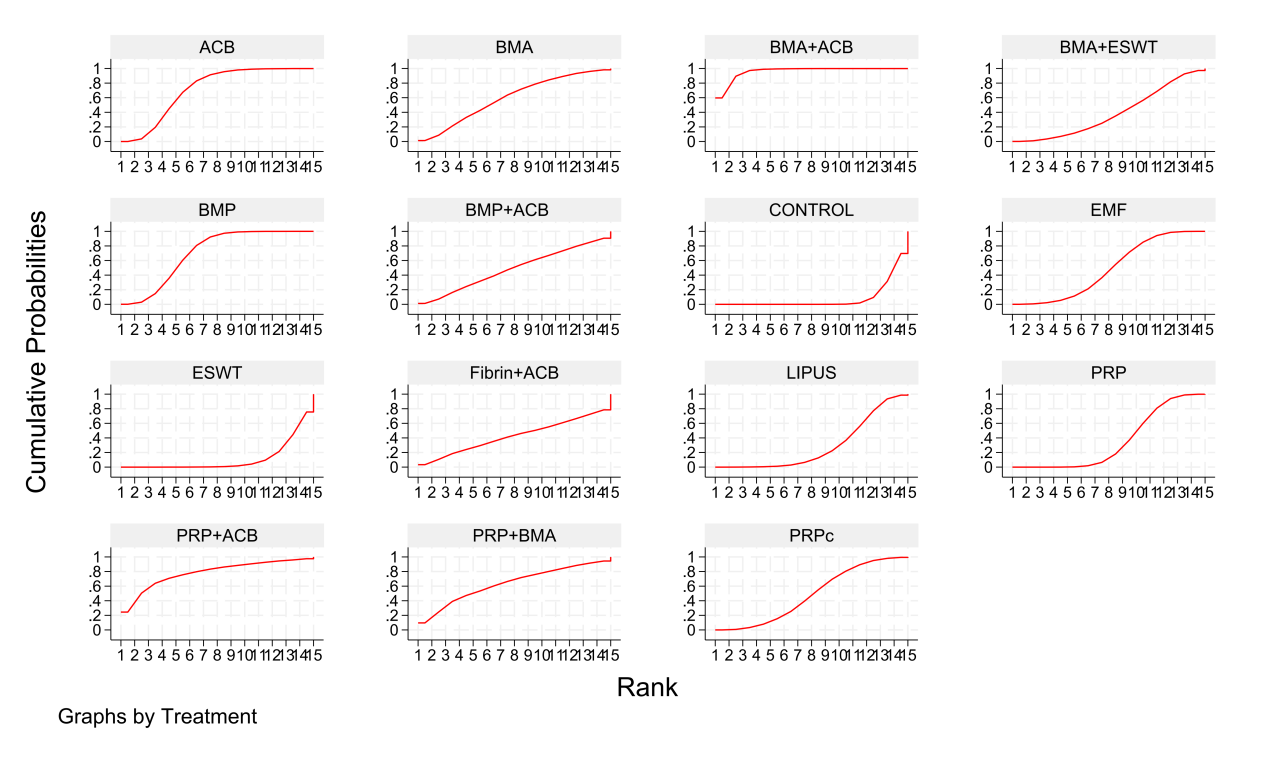


Fig.3 Probability Ranking Plot for healing rate

| Treatment | SUCRA | PrBest | MeanRank |

|------------+-------+--------+----------|

| CONTROL | 8.0 | 0.0 | 13.9 |

| PRP | 35.6 | 0.0 | 10.0 |

| ESWT | 11.3 | 0.0 | 13.4 |

| BMA | 59.7 | 1.3 | 6.6 |

| EMF | 48.7 | 0.0 | 8.2 |

| BMP | 70.2 | 0.1 | 5.2 |

| LIPUS | 29.2 | 0.0 | 10.9 |

| ACB | 71.6 | 0.0 | 5.0 |

| PRP+ACB | 78.2 | 24.5 | 4.1 |

| PRP+BMA | 63.4 | 9.6 | 6.1 |

| BMA+ESWT | 38.8 | 0.1 | 9.6 |

| PRPc | 48.6 | 0.0 | 8.2 |

| BMP+ACB | 48.4 | 1.2 | 8.2 |

| BMA+ACB | 96.0 | 59.7 | 1.6 |

| Fibrin+ACB | 42.4 | 3.4 | 9.1 |

**5.2 Results of the long bone subgroup for healing time**

**
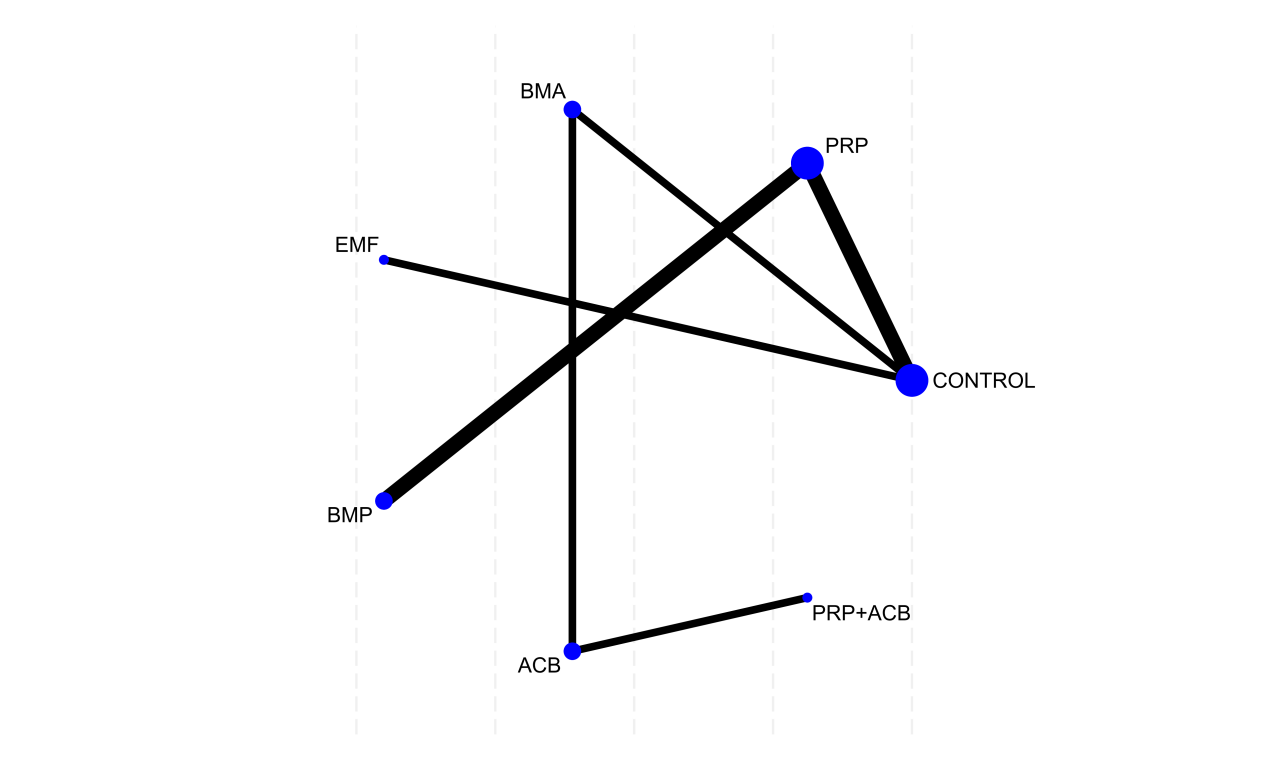
**

Fig.1 Network plot of results

**
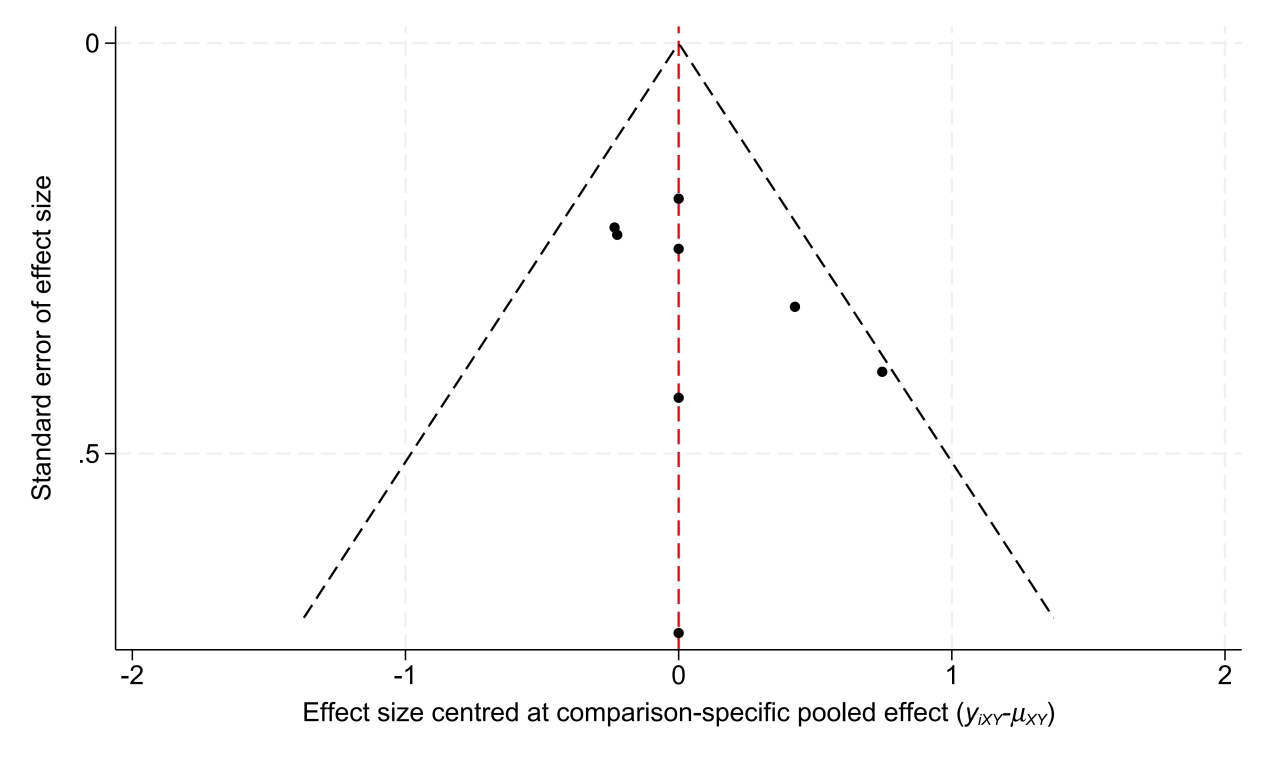
**

Fig.2 Funnel plot for healing time


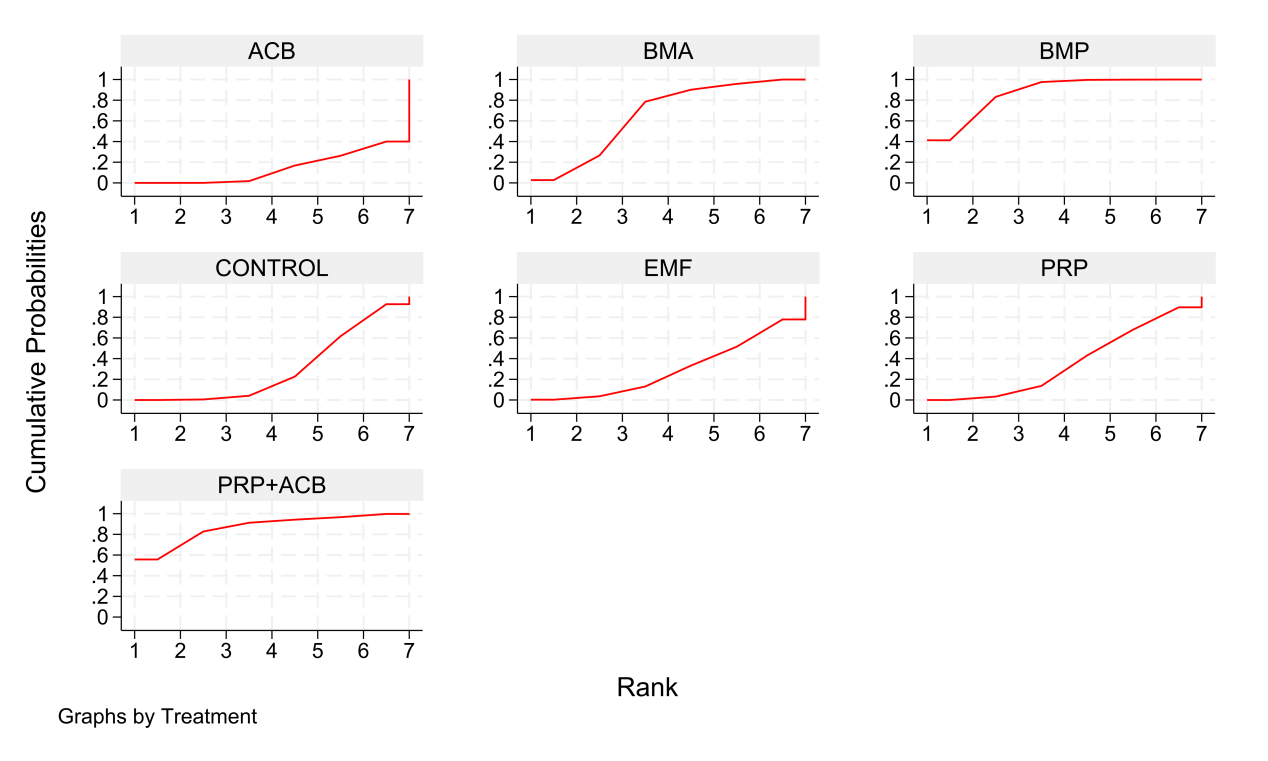


Fig.3 Probability Ranking Plot for healing time

| Treatm~t | SUCRA | PrBest | MeanRank |

|----------+-------+--------+----------|

| CONTROL | 30.3 | 0.0 | 5.2 |

| PRP | 36.3 | 0.0 | 4.8 |

| BMA | 65.6 | 2.7 | 3.1 |

| EMF | 30.0 | 0.3 | 5.2 |

| BMP | 86.9 | 41.3 | 1.8 |

| ACB | 14.2 | 0.0 | 6.2 |

| PRP+ACB | 86.7 | 55.7 | 1.8 |
