# Supplementary material for: Efficacy and safety of biological agents and physical therapies for delayed union or nonunion of fractures: a network meta-analysis of randomized controlled trials
Source: BMC Musculoskelet Disord. 2026 May 28;27:637. doi: 10.1186/s12891-026-09973-w (PMC13422364; doi:10.1186/s12891-026-09973-w)
Supplement: Supplementary file 2 — Supplementary Material 2. [file 12891_2026_9973_MOESM2_ESM.pdf]

Comparison	Number of studies	Within-study bias	Reporting bias	Indirectness	Imprecision	Heterogeneity	Incoherence	Confidence rating
**Mixed evidence**								
1:02	2	Some concerns	Low risk	No concerns	Major concerns	No concerns	No concerns	Low
1:03	1	Major concerns	Low risk	No concerns	Major concerns	No concerns	Some concerns	Low
1:04	1	Major concerns	Low risk	No concerns	Major concerns	No concerns	No concerns	Low
1:05	6	Some concerns	Low risk	No concerns	No concerns	Major concerns	Some concerns	Low
1:07	3	Some concerns	Low risk	No concerns	Major concerns	No concerns	Some concerns	Low
1:11	1	Major concerns	Low risk	No concerns	Major concerns	No concerns	Some concerns	Low
2:06	2	Major concerns	Low risk	No concerns	Major concerns	No concerns	No concerns	Low
4:08	2	Major concerns	Low risk	No concerns	Major concerns	No concerns	No concerns	Low
6:08	2	Some concerns	Low risk	No concerns	Major concerns	No concerns	No concerns	Low
10:08	1	Major concerns	Low risk	No concerns	Major concerns	No concerns	Some concerns	Low
13:03	1	Major concerns	Low risk	No concerns	Major concerns	No concerns	Some concerns	Low
15:02	1	No concerns	Low risk	No concerns	Major concerns	No concerns	Some concerns	Low
17:08	3	Major concerns	Low risk	No concerns	Major concerns	No concerns	Some concerns	Low
18:08	1	Major concerns	Low risk	No concerns	Major concerns	No concerns	Some concerns	Low
19:08	1	No concerns	Low risk	No concerns	Major concerns	No concerns	Some concerns	Low
**Indirect evidence**								
1:06	0	Major concerns	Low risk	No concerns	No concerns	Major concerns	Some concerns	Low
1:08	0	Major concerns	Low risk	No concerns	No concerns	Major concerns	Some concerns	Low
1:10	0	Major concerns	Low risk	No concerns	No concerns	Major concerns	Some concerns	Low
1:13	0	Major concerns	Low risk	No concerns	Major concerns	No concerns	Some concerns	Low
1:15	0	Some concerns	Low risk	No concerns	Major concerns	No concerns	Some concerns	Low
1:17	0	Major	Low risk	No concerns	No	Major	Some	Low

Comparison	Number of studies	Within-study bias	Reporting bias	Indirectness	Imprecision	Heterogeneity	Incoherence	Confidence rating
		concerns			concerns	concerns	concerns	
1:18	0	Major concerns	Low risk	No concerns	No concerns	No concerns	Some concerns	Low
1:19	0	Some concerns	Low risk	No concerns	Major concerns	No concerns	Some concerns	Low
2:03	0	Major concerns	Low risk	No concerns	Major concerns	No concerns	Some concerns	Low
2:04	0	Major concerns	Low risk	No concerns	Major concerns	No concerns	Some concerns	Low
2:05	0	Some concerns	Low risk	No concerns	Major concerns	No concerns	Some concerns	Low
2:07	0	Some concerns	Low risk	No concerns	Major concerns	No concerns	Some concerns	Low
2:08	0	Major concerns	Low risk	No concerns	Major concerns	No concerns	Some concerns	Low
10:02	0	Major concerns	Low risk	No concerns	Major concerns	No concerns	Some concerns	Low
11:02	0	Major concerns	Low risk	No concerns	Major concerns	No concerns	Some concerns	Low
13:02	0	Major concerns	Low risk	No concerns	Major concerns	No concerns	Some concerns	Low
17:02	0	Major concerns	Low risk	No concerns	Major concerns	No concerns	Some concerns	Low
18:02	0	Major concerns	Low risk	No concerns	No concerns	Major concerns	Some concerns	Low
19:02	0	Some concerns	Low risk	No concerns	Major concerns	No concerns	Some concerns	Low
3:04	0	Major concerns	Low risk	No concerns	Major concerns	No concerns	Some concerns	Low
3:05	0	Some concerns	Low risk	No concerns	Major concerns	No concerns	Some concerns	Low
3:06	0	Major concerns	Low risk	No concerns	Major concerns	No concerns	Some concerns	Low
3:07	0	Some concerns	Low risk	No concerns	Major concerns	No concerns	Some concerns	Low
3:08	0	Major concerns	Low risk	No concerns	Major concerns	No concerns	Some concerns	Low
10:03	0	Major concerns	Low risk	No concerns	Major concerns	No concerns	Some concerns	Low
11:03	0	Major concerns	Low risk	No concerns	Major concerns	No concerns	Some concerns	Low
15:03	0	Some concerns	Low risk	No concerns	Major concerns	No concerns	Some concerns	Low
17:03	0	Major concerns	Low risk	No concerns	Major concerns	No concerns	Some concerns	Low
18:03	0	Major	Low risk	No concerns	Major	No concerns	Some	Low

Comparison	Number of studies	Within-study bias	Reporting bias	Indirectness	Imprecision	Heterogeneity	Incoherence	Confidence rating
		concerns			concerns		concerns	
19:03	0	Some concerns	Low risk	No concerns	Major concerns	No concerns	Some concerns	Low
4:05	0	Some concerns	Low risk	No concerns	Major concerns	No concerns	Some concerns	Low
4:06	0	Major concerns	Low risk	No concerns	Major concerns	No concerns	Some concerns	Low
4:07	0	Some concerns	Low risk	No concerns	Major concerns	No concerns	Some concerns	Low
10:04	0	Major concerns	Low risk	No concerns	Major concerns	No concerns	Some concerns	Low
11:04	0	Major concerns	Low risk	No concerns	Major concerns	No concerns	Some concerns	Low
13:04	0	Major concerns	Low risk	No concerns	Major concerns	No concerns	Some concerns	Low
15:04	0	Some concerns	Low risk	No concerns	Major concerns	No concerns	Some concerns	Low
17:04	0	Major concerns	Low risk	No concerns	Major concerns	No concerns	Some concerns	Low
18:04	0	Major concerns	Low risk	No concerns	Major concerns	No concerns	Some concerns	Low
19:04	0	Some concerns	Low risk	No concerns	Major concerns	No concerns	Some concerns	Low
5:06	0	Some concerns	Low risk	No concerns	Major concerns	No concerns	Some concerns	Low
5:07	0	Some concerns	Low risk	No concerns	Major concerns	No concerns	Some concerns	Low
5:08	0	Some concerns	Low risk	No concerns	Major concerns	No concerns	Some concerns	Low
10:05	0	Major concerns	Low risk	No concerns	Major concerns	No concerns	Some concerns	Low
11:05	0	Some concerns	Low risk	No concerns	Major concerns	No concerns	Some concerns	Low
13:05	0	Major concerns	Low risk	No concerns	Major concerns	No concerns	Some concerns	Low
15:05	0	Some concerns	Low risk	No concerns	Major concerns	No concerns	Some concerns	Low
17:05	0	Major concerns	Low risk	No concerns	Major concerns	No concerns	Some concerns	Low
18:05	0	Major concerns	Low risk	No concerns	Major concerns	No concerns	Some concerns	Low
19:05	0	Some concerns	Low risk	No concerns	Major concerns	No concerns	Some concerns	Low
6:07	0	Some concerns	Low risk	No concerns	Major concerns	No concerns	Some concerns	Low
10:06	0	Major	Low risk	No concerns	Major	No concerns	Some	Low

Comparison	Number of studies	Within-study bias	Reporting bias	Indirectness	Imprecision	Heterogeneity	Incoherence	Confidence rating
		concerns			concerns		concerns	
11:06	0	Major concerns	Low risk	No concerns	Major concerns	No concerns	Some concerns	Low
13:06	0	Major concerns	Low risk	No concerns	Major concerns	No concerns	Some concerns	Low
15:06	0	Some concerns	Low risk	No concerns	Major concerns	No concerns	Some concerns	Low
17:06	0	Major concerns	Low risk	No concerns	Major concerns	No concerns	Some concerns	Low
18:06	0	Major concerns	Low risk	No concerns	Major concerns	No concerns	Some concerns	Low
19:06	0	Some concerns	Low risk	No concerns	Major concerns	No concerns	Some concerns	Low
7:08	0	Some concerns	Low risk	No concerns	Major concerns	No concerns	Some concerns	Low
10:07	0	Major concerns	Low risk	No concerns	Major concerns	No concerns	Some concerns	Low
11:07	0	Some concerns	Low risk	No concerns	Major concerns	No concerns	Some concerns	Low
13:07	0	Major concerns	Low risk	No concerns	Major concerns	No concerns	Some concerns	Low
15:07	0	Some concerns	Low risk	No concerns	Major concerns	No concerns	Some concerns	Low
17:07	0	Major concerns	Low risk	No concerns	Major concerns	No concerns	Some concerns	Low
18:07	0	Major concerns	Low risk	No concerns	No concerns	Major concerns	Some concerns	Low
19:07	0	Some concerns	Low risk	No concerns	Major concerns	No concerns	Some concerns	Low
11:08	0	Major concerns	Low risk	No concerns	Major concerns	No concerns	Some concerns	Low
13:08	0	Major concerns	Low risk	No concerns	Major concerns	No concerns	Some concerns	Low
15:08	0	Some concerns	Low risk	No concerns	Major concerns	No concerns	Some concerns	Low
10:11	0	Major concerns	Low risk	No concerns	Major concerns	No concerns	Some concerns	Low
10:13	0	Major concerns	Low risk	No concerns	Major concerns	No concerns	Some concerns	Low
10:15	0	Some concerns	Low risk	No concerns	Major concerns	No concerns	Some concerns	Low
10:17	0	Major concerns	Low risk	No concerns	Major concerns	No concerns	Some concerns	Low
10:18	0	Major concerns	Low risk	No concerns	Major concerns	No concerns	Some concerns	Low
10:19	0	Some	Low risk	No concerns	Major	No concerns	Some	Low

Comparison	Number of studies	Within-study bias	Reporting bias	Indirectness	Imprecision	Heterogeneity	Incoherence	Confidence rating
		concerns			concerns		concerns	
11:13	0	Major concerns	Low risk	No concerns	Major concerns	No concerns	Some concerns	Low
11:15	0	Some concerns	Low risk	No concerns	Major concerns	No concerns	Some concerns	Low
11:17	0	Major concerns	Low risk	No concerns	Major concerns	No concerns	Some concerns	Low
11:18	0	Major concerns	Low risk	No concerns	Major concerns	No concerns	Some concerns	Low
11:19	0	Some concerns	Low risk	No concerns	Major concerns	No concerns	Some concerns	Low
13:15	0	Some concerns	Low risk	No concerns	Major concerns	No concerns	Some concerns	Low
13:17	0	Major concerns	Low risk	No concerns	Major concerns	No concerns	Some concerns	Low
13:18	0	Major concerns	Low risk	No concerns	Major concerns	No concerns	Some concerns	Low
13:19	0	Some concerns	Low risk	No concerns	Major concerns	No concerns	Some concerns	Low
15:17	0	Some concerns	Low risk	No concerns	Major concerns	No concerns	Some concerns	Low
15:18	0	Some concerns	Low risk	No concerns	Major concerns	No concerns	Some concerns	Low
15:19	0	Some concerns	Low risk	No concerns	Major concerns	No concerns	Some concerns	Low
17:18	0	Major concerns	Low risk	No concerns	Major concerns	No concerns	Some concerns	Low
17:19	0	Some concerns	Low risk	No concerns	Major concerns	No concerns	Some concerns	Low
18:19	0	Some concerns	Low risk	No concerns	Major concerns	No concerns	Some concerns	Low

Comparison	Number of studies	Within-study bias	Reporting bias	Indirectness	Imprecision	Heterogeneity	Incoherence	Confidence rating
Mixed evidence								
1:02	1	No concerns	Low risk	No concerns	Major concerns	No concerns	No concerns	Low
2:06	1	Major concerns	Low risk	No concerns	Major concerns	No concerns	No concerns	Low
4:08	1	Major concerns	Low risk	No concerns	Major concerns	No concerns	No concerns	Low
6:08	2	Some concerns	Low risk	No concerns	No concerns	Major concerns	No concerns	Low
18:08	1	Major concerns	Low risk	No concerns	No concerns	Major concerns	No concerns	Low
Indirect evidence								
1:04	0	Some concerns	Low risk	No concerns	Major concerns	No concerns	No concerns	Low
1:06	0	Some concerns	Low risk	No concerns	Major concerns	No concerns	No concerns	Low
1:08	0	Some concerns	Low risk	No concerns	No concerns	Major concerns	No concerns	Low
1:18	0	Some concerns	Low risk	No concerns	Major concerns	No concerns	No concerns	Low
2:04	0	Major concerns	Low risk	No concerns	Major concerns	No concerns	No concerns	Low
2:08	0	Major concerns	Low risk	No concerns	Major concerns	No concerns	No concerns	Low
18:02	0	Major concerns	Low risk	No concerns	Major concerns	No concerns	No concerns	Low
4:06	0	Major concerns	Low risk	No concerns	Major concerns	No concerns	No concerns	Low
18:04	0	Major concerns	Low risk	No concerns	Major concerns	No concerns	No concerns	Low
18:06	0	Major concerns	Low risk	No concerns	Major concerns	No concerns	No concerns	Low

Comparison	Number of studies	Within-study bias	Reporting bias	Indirectness	Imprecision	Heterogeneity	Incoherence	Confidence rating
Mixed evidence								
1:02	2	Some concerns	Low risk	No concerns	Major concerns	No concerns	Major concerns	Low
1:04	1	Major concerns	Low risk	No concerns	Major concerns	No concerns	Major concerns	Low
1:05	1	Some concerns	Low risk	No concerns	Major concerns	No concerns	Major concerns	High
1:07	1	Some concerns	Low risk	No concerns	No concerns	No concerns	Major concerns	Low
2:06	2	Major concerns	Low risk	No concerns	No concerns	Major concerns	Major concerns	Low
4:08	1	Major concerns	Low risk	No concerns	No concerns	Major concerns	Major concerns	Low
10:08	1	Major concerns	Low risk	No concerns	No concerns	Major concerns	Major concerns	Low
Indirect evidence								
1:06	0	Some concerns	Low risk	No concerns	No concerns	Major concerns	Major concerns	Low
1:08	0	Major concerns	Low risk	No concerns	Major concerns	No concerns	Major concerns	Low
1:10	0	Major concerns	Low risk	No concerns	Major concerns	No concerns	Major concerns	Low
2:04	0	Some concerns	Low risk	No concerns	Major concerns	No concerns	Major concerns	Low
2:05	0	Some concerns	Low risk	No concerns	Major concerns	No concerns	Major concerns	Low
2:07	0	Some concerns	Low risk	No concerns	No concerns	No concerns	Major concerns	Low
2:08	0	Major concerns	Low risk	No concerns	Major concerns	No concerns	Major concerns	Low
10:02	0	Major concerns	Low risk	No concerns	Major concerns	No concerns	Major concerns	Low
4:05	0	Major concerns	Low risk	No concerns	Major concerns	No concerns	Major concerns	Low
4:06	0	Major concerns	Low risk	No concerns	Major concerns	No concerns	Major concerns	Low
4:07	0	Major concerns	Low risk	No concerns	No concerns	No concerns	Major concerns	Low

Comparison	Number of studies	Within-study bias	Reporting bias	Indirectness	Imprecision	Heterogeneity	Incoherence	Confidence rating
10:04	0	Major concerns	Low risk	No concerns	Major concerns	No concerns	Major concerns	Low
5:06	0	Some concerns	Low risk	No concerns	No concerns	Major concerns	Major concerns	Low
5:07	0	Some concerns	Low risk	No concerns	No concerns	No concerns	Major concerns	Low
5:08	0	Major concerns	Low risk	No concerns	Major concerns	No concerns	Major concerns	Low
10:05	0	Major concerns	Low risk	No concerns	Major concerns	No concerns	Major concerns	Low
6:07	0	Some concerns	Low risk	No concerns	No concerns	No concerns	Major concerns	Low
6:08	0	Major concerns	Low risk	No concerns	No concerns	Major concerns	Major concerns	Low
10:06	0	Major concerns	Low risk	No concerns	Major concerns	No concerns	Major concerns	Low
7:08	0	Major concerns	Low risk	No concerns	No concerns	No concerns	Major concerns	Low
10:07	0	Major concerns	Low risk	No concerns	No concerns	Major concerns	Major concerns	Low
